# Sphingosine 1-phosphate receptor 1 signaling in macrophages reduces atherosclerosis in LDL receptor–deficient mice

**DOI:** 10.1172/jci.insight.158127

**Published:** 2024-11-12

**Authors:** Francesco Potì, Enrica Scalera, Renata Feuerborn, Josephine Fischer, Lilli Arndt, Georg Varga, Evangelia Pardali, Matthias D. Seidl, Manfred Fobker, Gerhard Liebisch, Bettina Hesse, Alexander H. Lukasz, Jan Rossaint, Beate E. Kehrel, Frank Rosenbauer, Thomas Renné, Christina Christoffersen, Manuela Simoni, Ralph Burkhardt, Jerzy-Roch Nofer

**Affiliations:** 1 Unit of Neuroscience, Department of Medicine and Surgery, University of Parma, Parma, Italy.; 2 Unit of Endocrinology, Department of Biomedical, Metabolic and Neural Sciences, University of Modena and Reggio Emilia, Italy.; 3 Department of Food and Drug, University of Parma, Parma, Italy.; 4 Central Laboratory Facility, University Hospital Münster, Münster, Germany.; 5 Institute of Molecular Tumor Biology, University of Münster, Münster, Germany.; 6 Institute for Laboratory Medicine, Clinical Chemistry and Molecular Diagnostics, University of Leipzig, Germany.; 7 Department of Pediatric Rheumatology and Immunology, University Children’s Hospital Münster, Münster, Germany.; 8 Department of Cardiology, University Hospital Münster, Münster, Germany.; 9 Pharvaris GmbH, Zug, Switzerland.; 10 Institute of Pharmacology and Toxicology, University of Münster, Münster, Germany.; 11 Institute of Clinical Chemistry and Laboratory Medicine, University Hospital Regensburg, Regensburg, Germany.; 12 Division of General Internal Medicine, Nephrology, and Rheumatology, Department of Medicine D, and; 13 Department of Anesthesiology, Intensive Care and Pain Medicine, University Hospital Münster, Münster, Germany.; 14 Institute of Clinical Chemistry and Laboratory Medicine, University Medical Center Hamburg-Eppendorf, Hamburg, Germany.; 15 Irish Centre for Vascular Biology, School of Pharmacy and Biomolecular Sciences, Royal College of Surgeons in Ireland, Dublin, Ireland.; 16 Center for Thrombosis and Hemostasis (CTH), Johannes Gutenberg University Medical Center, Mainz, Germany.; 17 Department of Clinical Biochemistry, Rigshospitalet, and Department of Biomedical Sciences, University of Copenhagen, Copenhagen, Denmark.; 18 Institute for Laboratory Medicine, Marien-Hospital, Niels-Stensen-Kliniken, Osnabrück, Germany.

**Keywords:** Inflammation, Vascular biology, Atherosclerosis, Lipoproteins, Macrophages

## Abstract

Sphingosine 1-phosphate (S1P) is a lysosphingolipid with antiatherogenic properties, but mechanisms underlying its effects remain unclear. We here investigated atherosclerosis development in cholesterol-rich diet–fed LDL receptor–deficient mice with high or low overexpression levels of S1P receptor 1 (S1P_1_) in macrophages. S1P_1_-overexpressing macrophages showed increased activity of transcription factors PU.1, interferon regulatory factor 8 (IRF8), and liver X receptor (LXR) and were skewed toward an M2-distinct phenotype characterized by enhanced production of IL-10, IL-1RA, and IL-5; increased ATP-binding cassette transporter A1– and G1–dependent cholesterol efflux; increased expression of MerTK and efferocytosis; and reduced apoptosis due to elevated B cell lymphoma 6 and Maf bZIP B. A similar macrophage phenotype was observed in mice administered S1P_1_-selective agonist KRP203. Mechanistically, the enhanced PU.1, IRF8, and LXR activity in S1P_1_-overexpressing macrophages led to downregulation of the cAMP-dependent PKA and activation of the signaling cascade encompassing protein kinases AKT and mTOR complex 1 as well as the late endosomal/lysosomal adaptor MAPK and mTOR activator 1. Atherosclerotic lesions in aortic roots and brachiocephalic arteries were profoundly or moderately reduced in mice with high and low S1P_1_ overexpression in macrophages, respectively. We conclude that S1P_1_ signaling polarizes macrophages toward an antiatherogenic functional phenotype and countervails the development of atherosclerosis in mice.

## Abstract 2

Sphingosine-1-phosphate (S1P) signaling mediated by S1P receptor type 1 polarizes macrophages towards an anti-atherogenic functional phenotype and countervails the development of atherosclerosis in mice.

## Graphical abstract

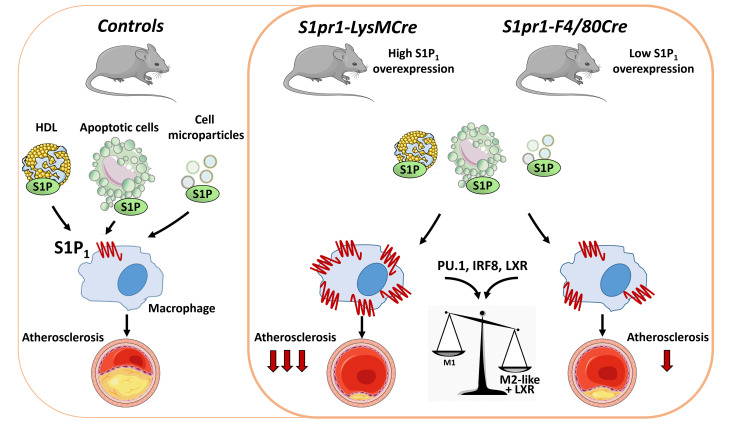

## Introduction

Sphingosine 1-phosphate (S1P) is a membrane-derived lysosphingolipid with multiple regulatory roles in physiology (1–3). Formation of S1P from sphingosine is catalyzed by sphingosine kinases (SK1 and SK2), and its breakdown to sphingosine or to 2-hexadecenal and phosphoethanolamine is mediated by S1P phosphatase or lyase, respectively (1–3). The S1P formed by SKs either binds to intracellular target proteins or is exported from cells to act as a ligand of 5 S1P-specific G protein–coupled receptors (S1P_1–5_) (2, 3). The physiological effects associated with S1P receptor activation are determined by the specific G protein coupling. S1P_1_ couples with the inhibitory G protein α subunit (G_αi_), which inhibits adenylate cyclase and lowers intracellular cAMP. In addition, G_i_ activates kinases such as AKT, PKCα and ε, and ERK1/2 (2–4). The signaling through S1P_1_ promotes beneficial processes in the vasculature, including endothelial proliferation and survival, endothelial barrier stabilization, and amelioration of endothelial dysfunction (2, 3, 5). Furthermore, acting via S1P_1_, S1P interferes with proliferation, migration, and activation of lymphocytes and monocytes/macrophages and regulates their recruitment to inflamed tissues (3, 6). These modulatory activities explain the aggravation of inflammation in S1P_1_ deficiency (7, 8) and account for favorable effects exerted by S1P_1_ agonists in animal models of inflammatory diseases (2, 3, 9).

Erythrocytes, platelets, and endothelial cells are the major sources of S1P in plasma, in which it binds to albumin or apolipoprotein M (apoM) found in a subpopulation of HDL (9, 10). This apoM^+^ HDL-bound S1P was suggested to contribute to atheroprotective effects attributed to HDL in epidemiological studies (10–12). S1P in plasma correlates with HDL in a concentration range, in which this lipoprotein protects against atherosclerosis, and lower levels of both, total and HDL-bound S1P, were noted in patients with coronary artery disease (13–15). However, studies on the effects of S1P on atherosclerosis in animal models led to disparate results. Synthetic S1P mimetics such as FTY720 — an S1P_1,3,4,5_ pan-agonist — and KRP203 — a specific S1P_1_ agonist — diminished lesions in LDL receptor–deficient (*Ldlr*^–/–^) models, improved endothelial function, and suppressed macrophage activation (16, 17). Likewise, elevating S1P levels by eliminating SK2 or S1P lyase in hematogenous cells blunted atherosclerosis in *Ldlr*^–/–^ mice (18, 19). Conversely, *S1pr1* deletion in endothelial cells or macrophages enhanced plaque development in mice (8, 20). By contrast, reduced atherosclerotic lesion formation was observed in apolipoprotein E–deficient (*Apoe*^–/–^) mice deficient in *S1pr2* (21). Moreover, S1P elevation in *Apoe*^–/–^ mice overexpressing apoM augmented aortic root but not aortic arch atherosclerosis, and this effect was abolished in uremia (22). Thus, the involvement of S1P in atherosclerosis depends on the S1P receptor involved, the location of lesions, and the experimental setting. In the present study, we attempted to resolve the controversy over the identity of S1P receptors and their cellular targets mediating atheroprotective effects. Toward this aim, we generated mouse lines with high or low overexpression levels of S1P_1_ in macrophages. Our results demonstrate that the amplification of S1P_1_ signaling produces a distinct subset of M2-like macrophages with antiinflammatory and antiatherogenic properties and ameliorates atherosclerosis development in *Ldlr*^–/–^ mice. Since atheroprotective effects related to S1P_1_ overexpression were preserved in *Ldlr*^–/–^ mice lacking apoM, our findings suggest that at least with respect to macrophages S1P binding to HDL is redundant for its beneficial effects in atherosclerosis.

## Results

### S1pr1-LysMCre and S1pr1-F4/80Cre mice overexpress S1P_1_ in macrophages.

To amplify S1P_1_ signaling in target cells, double-transgenic mice expressing mouse S1P_1_ in mononuclear cells were constructed by crossing 2 lines. The *S1pr1-KI* line carries a transgenic cassette in the Rosa 26 locus harboring the mouse *S1pr1* cDNA and separated from the CAG promoter by a lox-Stop-lox insert (Supplemental Figure 1A; supplemental material available online with this article; https://doi.org/10.1172/jci.insight.158127DS1). *S1pr1-KI* mice were crossed to either *LysMCre* or *F4/80Cre* mice expressing Cre recombinase under the control of the lysozyme M or F4/80 promoter, respectively. In the offspring the lox-Stop-lox insert is excised in Cre-expressing cells, which induces cDNA expression driven by the CAG promoter. ImmGen Skyline tool for the lysozyme M promoter–driven Cre expression predicted highest activities in peritoneal macrophages (PMs) and neutrophils and slightly lower activities in tissue-resident macrophages (lung, adipose tissue, bone marrow) (Supplemental Figure 1B). With respect to the F4/80 promoter, the highest Cre expression predicted in PMs was 40% lower compared with the lysozyme M promoter, whereas Cre expressions predicted in tissue-resident macrophages were 70%–80% lower. No Cre expression was predicted for the F4/80 promoter in neutrophils and for both lysozyme M and F4/80 promoters in T cells (Supplemental Figure 1B). Accordingly, we observed strongly enhanced *S1pr1* mRNA expression in PMs and neutrophils from *S1pr1-LysMCre* mice and a weaker expression in *S1pr1-F4/80Cre* mice, whereas no differences were observed in T cells (Supplemental Figure 1C). In addition, a 3- to 6-fold increase in S1P_1_ on the macrophage or neutrophil cell surface was noted in double-transgenic lines and in the *S1pr1-LysMCre* line, respectively (Supplemental Figure 1C).

### S1P_1_ overexpression retards atherosclerosis and alters plaque composition.

**Figure 1:**
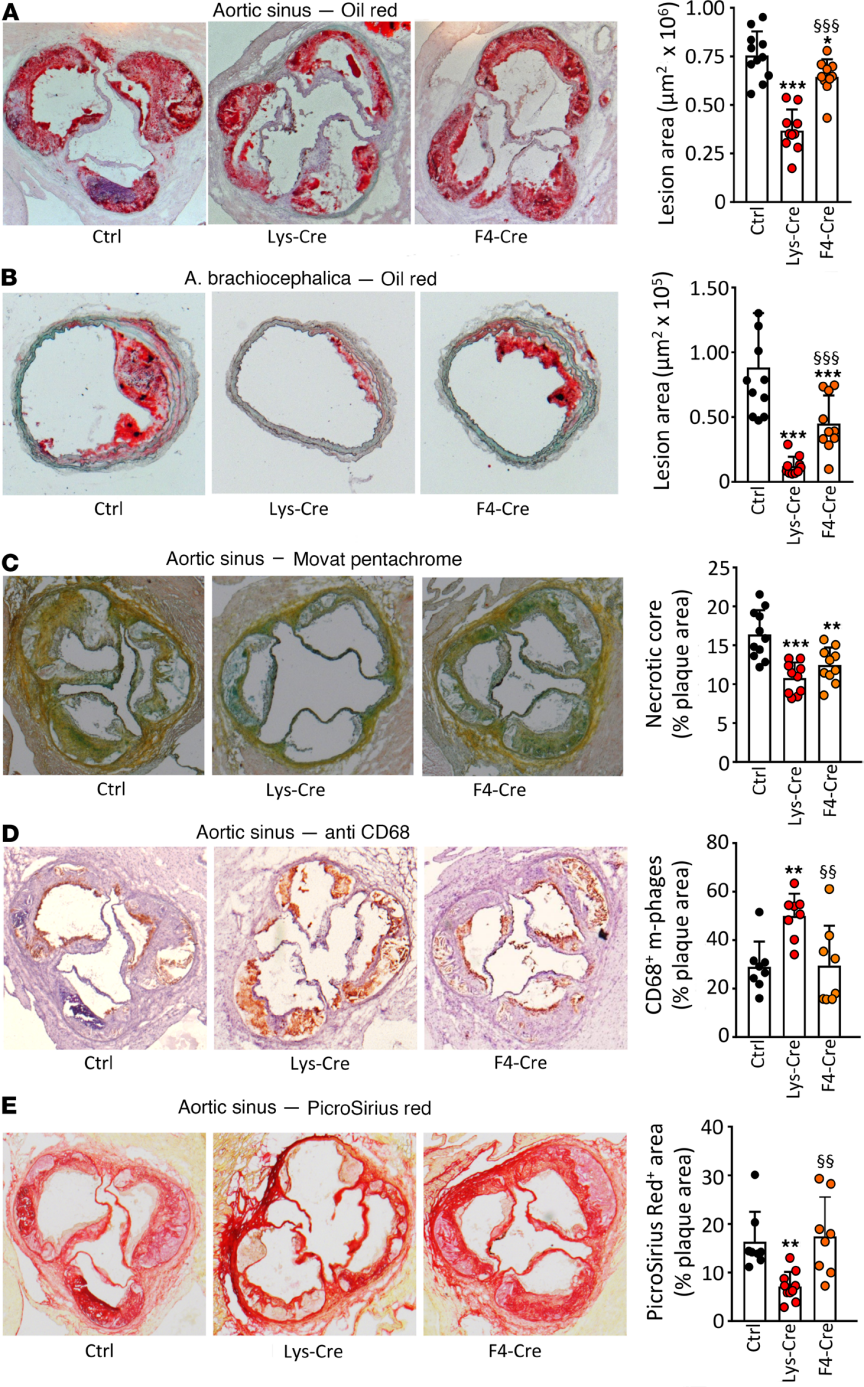
S1P_1_ overexpression in macrophages and monocytes retards atherosclerotic lesion development and alters plaque morphology in *Ldlr^–/–^* mice. Aortic root (**A** and **C**–**E**) and brachiocephalic arteries (**B**) from WD-fed *Ldlr*^–/–^ mice transplanted with *S1pr1-KI* (Ctrl, *n* = 11), *S1pr1-LysMCre* (Lys-Cre, *n* = 10), or *S1pr1-F4/80Cre* (F4-Cre, *n* = 10) BM were used for morphometry (**A** and **B**) or stained for necrotic core analysis (Movat pentachrome, **C**), macrophages (anti-CD68, **D**), or collagen (Picrosirius red, **E**). Bar graphs show the necrotic core extent or the macrophage or collagen content in plaques expressed as the percentage of lesion area. * - *P* < 0.05, ** - *P* < 0.01, *** - *P* < 0.001 (Lys-Cre vs. Ctrl or F4-Cre vs. Ctrl), ^§§^ - *P* < 0.01, ^§§§^ - *P* < 0.001 (Lys-Cre vs. F4-Cre, 1-way ANOVA except **B**: Kruskal-Wallis *h* test).

Atherosclerotic lesions were quantified in *Ldlr^–/–^* mice transplanted with *S1pr1-KI*, *S1pr1-LysMCre*, or *S1pr1-F4/80Cre* bone marrow (BM) and fed an atherogenic diet for 14 weeks. Lesion areas were profoundly reduced in aortic roots (~50%) and brachiocephalic arteries (~90%) in *S1pr1-LysMCre*–transplanted mice but only attenuated in aortic roots and roughly halved in brachiocephalic arteries from *S1pr1-F4/80Cre* chimeras (Figure 1, A and B). In addition, necrotic core area was significantly reduced, in both *S1pr1-LysMCre–* and *S1pr1-F4/80Cre*–transplanted mice (Figure 1C). Analysis of aortic root lesions yielded an increase of CD68-positive macrophage content in *S1pr1-LysMCre* but not *S1pr1-F4/80Cre* chimeras (Figure 1D). Conversely, the collagen content assessed by Picrosirius red staining was lower in *S1pr1-LysMCre–* but not *S1pr1-F4/80Cre–*transplanted mice (Figure 1E). The analysis of collagen-degrading proteases in PMs revealed no consistent mRNA expression pattern that could account for the reduced collagen amount in atherosclerotic plaques (Supplemental Figure 2).

### S1P_1_ overexpression affects leukocyte count but not body weight and plasma lipid profile.

Transplanted mice on Western diet (WD) showed expanded monocyte and B cell (B220^+^) and reduced T cell (CD3^+^) populations (Supplemental Table 1). Irrespective of dietary treatment, S1P_1_ overexpression controlled by the lysozyme M, but not the F4/80, promoter increased monocyte and neutrophil counts. Further, we observed reduced erythrocyte numbers, hematocrit, and hemoglobin in both *S1pr1-LysMCre* and *S1pr1-F4/80Cre* mice and decreased platelets in *S1pr1-LysMCre* mice on WD. Lymphocyte count and subpopulations were not affected. No differences in body weight and plasma lipid levels including S1P were observed between WD-fed *Ldlr^–/–^* mice transplanted with BM from *S1pr1-KI*, *S1pr1-LysMCre*, or *S1pr1-F4/80Cre* (Supplemental Table 2).

### S1P_1_ overexpression in macrophages activates PU.1 and interferon regulatory factor 8.

**Figure 2:**
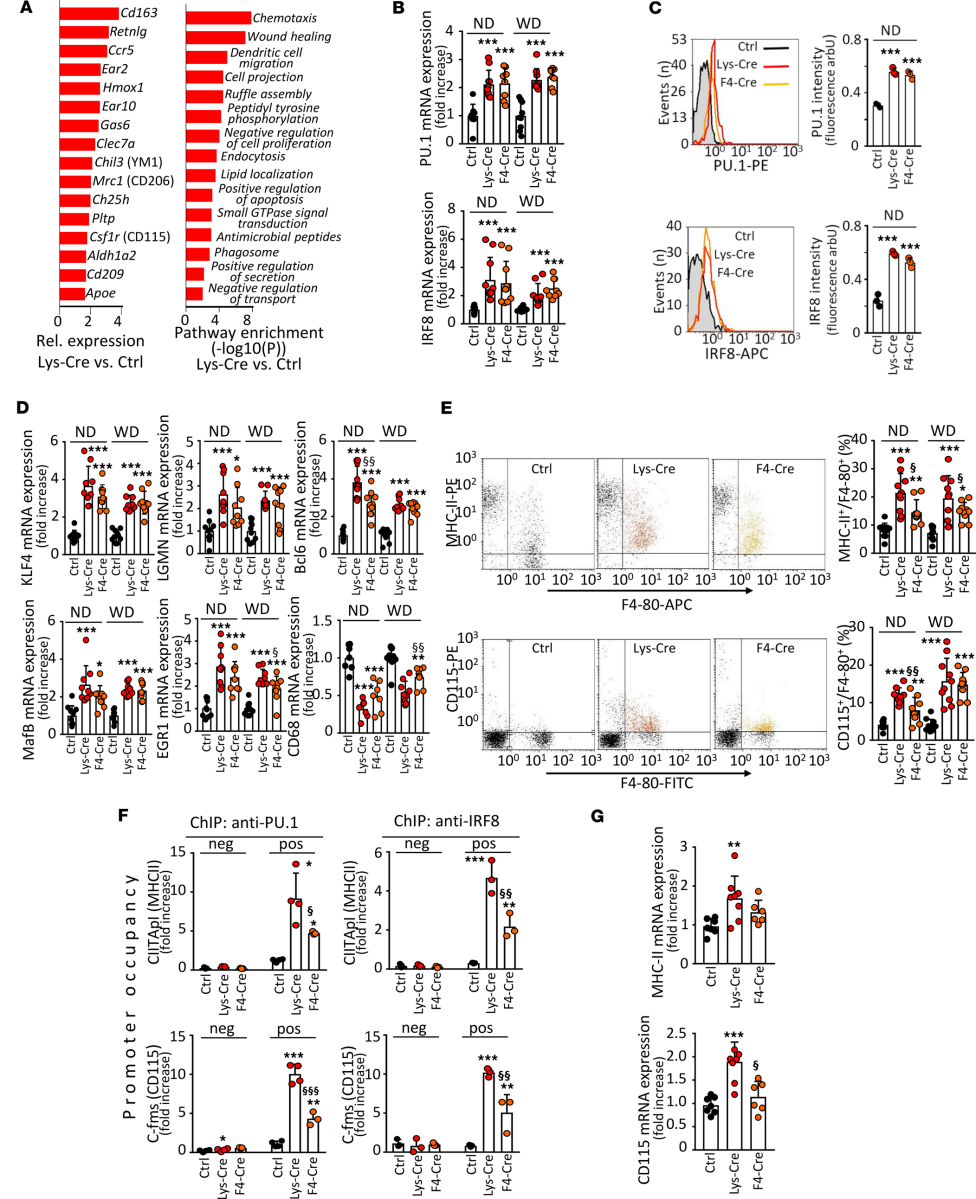
S1P_1_ overexpression in macrophages enhances expression and activation of PU.1 and interferon regulatory factor-8. PMs from either *S1pr1-KI* (Ctrl, *n* = 7–10), *S1pr1-LysMCre* (Lys-Cre, *n* = 7–10), or *S1pr1-F4/80Cre* (F4-Cre, *n* = 7–10) on normal diet (ND) or *Ldlr*^–/–^ mice transplanted with *S1pr1-KI* (*n* = 10), *S1pr1-LysMCre* (*n* = 9), or *S1pr1-F4/80Cre* (*n* = 9) BM on WD. (**A**) Gene expression in PMs from Lys-Cre and Ctrl mice (*n* = 3–4 for each group) assessed with microarrays. Left panel: expression pattern showing elevated genes controlled by PU.1/interferon regulatory factor-8 (IRF8) (colony-stimulating factor-1 receptor [*Csf1r*], *Clec7a*, *Mrc1*) and liver X receptor (LXR) (phospholipid transfer protein [*Pltp*], *Ch25h*, *Apoe*) in *S1pr1-LysMCre* mice. Right panel: enrichment analysis of upregulated transcripts in *S1pr1-LysMCre* mice. Chil3, chitinase 3-like; Hmox, heme oxygenase. (**B**) *Pu1* and *Irf8* expression by quantitative PCR (qPCR). mRNA normalized to *Gapdh* and shown relative to *S1pr1-KI*. (**C**) Intracellular stainings for PU.1 (top panels) and IRF8 (bottom panels) analyzed by flow cytometry (*n* = 3 for each group). (**D**) qPCR of PU.1 and IRF8 signature genes. (**E**) CD115 and MHC-II analyzed by flow cytometry. (**F**) PU.1 and IRF8 occupancy at *Cfms* (Cd115) and *Mhc2ta* (CIITApI, MHC-II) promoters analyzed by ChIP. Primers amplifying at –0.2 kb and +4.5 kb for *Cfms* and –74 bp and –3.0 kb for *CIITApI* used as positive binding sites and negative controls (*n* = 3–4 for each group). (**G**) CD115 and MHC-II mRNA expression in aortas of WD-fed *Ldlr^–/–^* mice receiving *S1pr1-KI*, *S1pr1-LysMCre*, or *S1pr1-F4/80Cre* BM. * - *P* < 0.05, ** - *P* < 0.01, *** - *P* < 0.001 (Lys-Cre vs. Ctrl or F4-Cre vs. Ctrl), ^§^ - *P* < 0.05, ^§§^ - *P* < 0.01, ^§§§^ - *P* < 0.001 (Lys-Cre vs. F4-Cre, 1-way or 2-way ANOVA except **B** IRF8 and **D** CIITApI/anti-PU.1: Kruskal-Wallis *h* test).

To characterize the transcriptional response to S1P_1_ overexpression, we performed gene expression analysis on PMs using oligonucleotide microarrays. mRNA expression levels of several genes involved in antiinflammatory macrophage polarization were increased in cells from *S1pr1-LysMCre* mice (Figure 2A). These findings were corroborated in a pathway and process enrichment analysis. Upregulated transcripts in macrophages from *S1pr1-LysMCre* mice revealed significant enrichment for antiinflammatory phenotype-associated functions such as wound healing (Figure 2A). Since several antiinflammatory genes identified in our analysis are directly or indirectly controlled by the transcription factors IRF8 and PU.1 (23, 24), we next assessed the effect of S1P_1_ overexpression on IRF8 and PU.1. Both mRNA and protein levels of *Irf8*/IRF8 and *Spi1*/PU.1 were increased in PMs from *S1pr1-LysMCre* and *S1pr1-F4/80Cre* mice (Figure 2, B and C). In addition, we detected elevated mRNA expression of several atheroprotective genes coordinately regulated by PU.1 and IRF8 (23) in cells overexpressing S1P_1_ (Figure 2D). By contrast, the mRNA expression of *Cd68*, which is negatively controlled by PU.1/IRF8 or PU.1/IRF4 complexes, was downregulated by S1P_1_ overexpression (Figure 2D). As IRF4 levels were not altered in PMs from *S1pr1-LysMCre* and *S1pr1-F4/80Cre* mice (not shown), we attribute this effect to IRF8. Notably, S1P_1_-overexpressing cells showed an increased surface presence of MHC class II (MHC-II) and CD115 (Figure 2E), whose promoters are canonical targets of PU.1. To investigate transcriptional activities of PU.1 and IRF8, we used chromatin immunoprecipitation (ChIP) to assess their binding to the class II transactivator (*Ciita* = *Mhc2ta*) and the *Csfmr* (= *Cd115*) gene promoters. Both PU.1 and IRF8 occupancies at – 0.2 kb and – 74 bp binding sites in *Mhc2ta* and *Cd115* promoters, respectively, were increased in PMs from *S1pr1-LysMCre* and *S1pr1-F4/80Cre* mice (Figure 2F). We also observed increased *Mhc2ta* and *Cd115* mRNA expression levels in aortas from these animals (Figure 2G).

### S1P_1_ overexpression promotes antiinflammatory macrophage phenotype.

**Figure 3:**
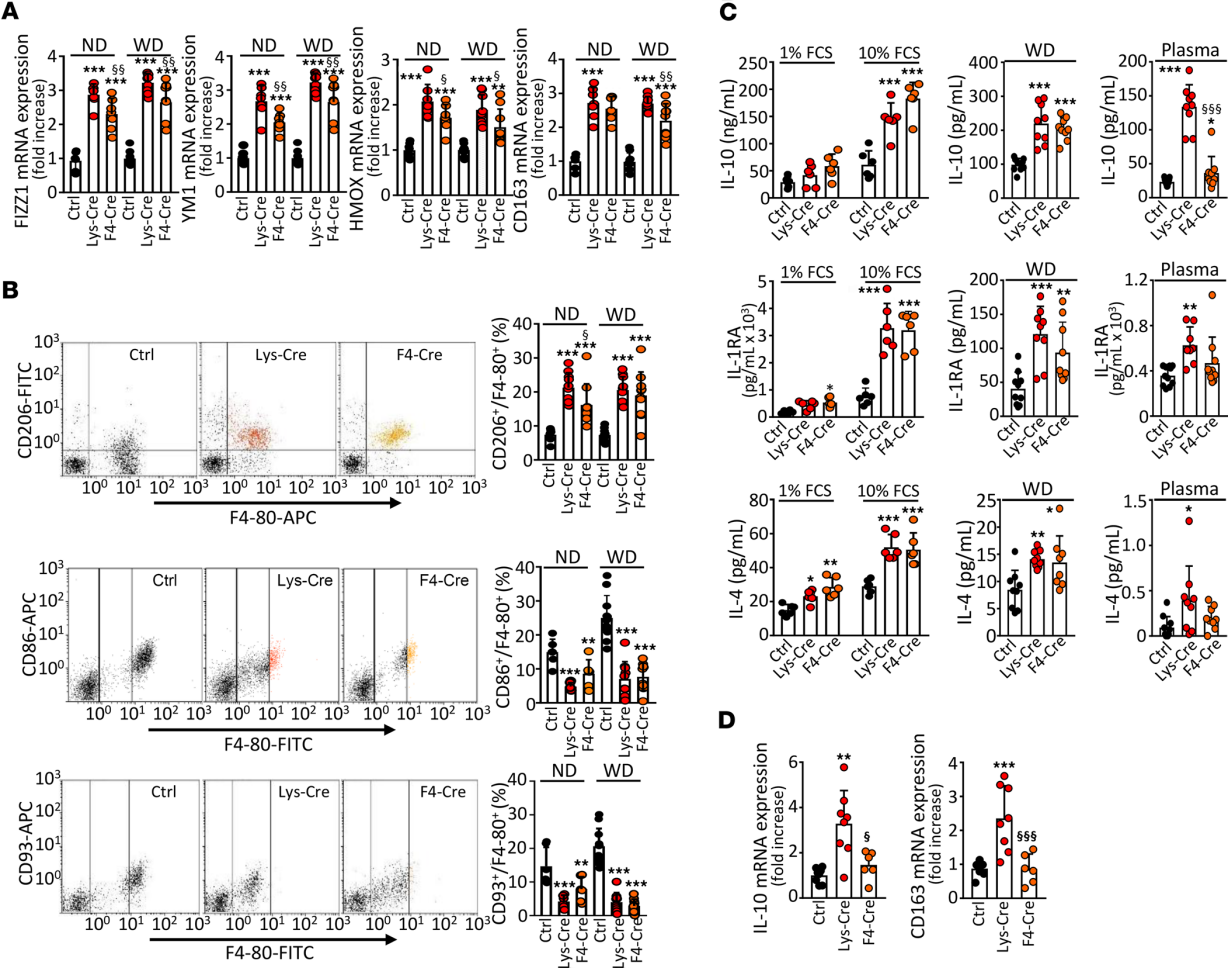
S1P_1_ overexpression in macrophages promotes antiinflammatory polarization. PMs from either *S1pr1-KI* (Ctrl, *n* = 6–10), *S1pr1-LysMCre* (Lys-Cre, *n* = 6–10), or *S1pr1-F4/80Cre* (F4-Cre, *n* = 6–9) on ND or *Ldlr^–/–^* mice transplanted with *S1pr1-KI* (*n* = 9–11), *S1pr1-LysMCre* (*n* = 9–10), or *S1pr1-F4/80Cre* (*n* = 9–10) BM on WD. (**A**) qPCR of antiinflammatory signature genes. mRNA normalized to *Gapdh* and presented relative to *S1pr1-KI*. (**B**) CD206 (antiinflammatory marker) and CD86 and CD93 (pro-inflammatory markers) analyzed by flow cytometry. (**C**) PMs incubated for 24 hours in media containing 1% FCS (ND-fed mice, *n* = 6 for each group, left panels) or 10% FCS (ND- and WD-fed mice, left and central panels). Cytokines in media and plasmas (WD-fed mice, *n* = 8–11 for each group, central and right panels) determined by ELISA. (**D**) Cytokine mRNA expression in aortas by qPCR (*n* = 6–8 for each group). * - *P* < 0.05, ** - *P* < 0.01, *** - *P* < 0.001 (Lys-Cre vs. Ctrl or F4-Cre vs. Ctrl, ^§^ - *P* < 0.05, ^§§^ - *P* < 0.01, ^§§§^ - *P* < 0.001 (Lys-Cre vs. F4-Cre, 1-way ANOVA except **C** IL-10/Plasma and **C** IL-4/Plasma: Kruskal-Wallis *h* test).

Transcription factors Krüppel-like factor 4 (KLF4) and Maf bZIP B (MAFB) control an antiinflammatory phenotype (25, 26). As mRNA expression levels of both, *Klf4* and *Mafb*, were elevated in S1P_1_-overexpressing PMs (Figure 2D), we next investigated macrophage polarization markers in S1P_1_-knockin mice. We detected enhanced mRNA expression of genes belonging to an antiinflammatory phenotype signature, including resistin-like α (*Retnla*, *Fizz1*), *Chi3l3* (*Ym1*), hemoglobin scavenger receptor (*Cd163*), and *Hmox1*, in *S1pr1-LysMCre* and *S1pr1-F4/80Cre* PMs (Figure 3A). In addition, these cells showed elevated cell surface presence of mannose receptor 1 (CD206; Figure 3B). By contrast, cell surface markers of pro-inflammatory macrophage phenotype CD86 and CD93 were downregulated in PMs overexpressing S1P_1_ (Figure 3B). To further elucidate the functional characteristics of *S1pr1-LysMCre* and *S1pr1-F4/80Cre* PMs, we measured antiinflammatory and pro-inflammatory cytokines in cell supernatants. Production of IL-10, IL-1RA, and IL-4 was increased in PMs from *S1pr1-LysMCre* and *S1pr1-F4/80Cre* mice (Figure 3C). In parallel, we detected elevated plasma levels of IL-10, IL-1RA, and IL-4 in *S1pr1-LysMCre* mice on WD (Figure 3C). Moreover, mRNA levels of *Cd163* and *Il10* were increased in the aortic arches of *Ldlr^–/–^* mice transplanted with *S1pr1-LysMCre* or *S1pr1-F4/80Cre* BM (Figure 3D). By contrast, production of pro-inflammatory cyto- and chemokines, including TNF-α, IL-6, KC1/GROα, and CCL5/RANTES, tended to be lower in S1P_1_-overexpressing PMs exposed for 24 hours to agonists of Toll-like receptor 2 (TLR-2; peptidoglycan [PGN], 0.02 μg/mL) or TLR-3 (polyinosinic–polycytidylic acid [pIC], 0.05 μg/mL; Supplemental Figure 3A). Concomitantly, lower levels of TNF-α, IL-6, KC1/GROα, and CCL5/RANTES were noted in plasma from *S1pr1-LysMCre* or *S1pr1-F4/80Cre* mice (Supplemental Figure 3B).

### S1P_1_ overexpression in macrophages activates LXR.

**Figure 4:**
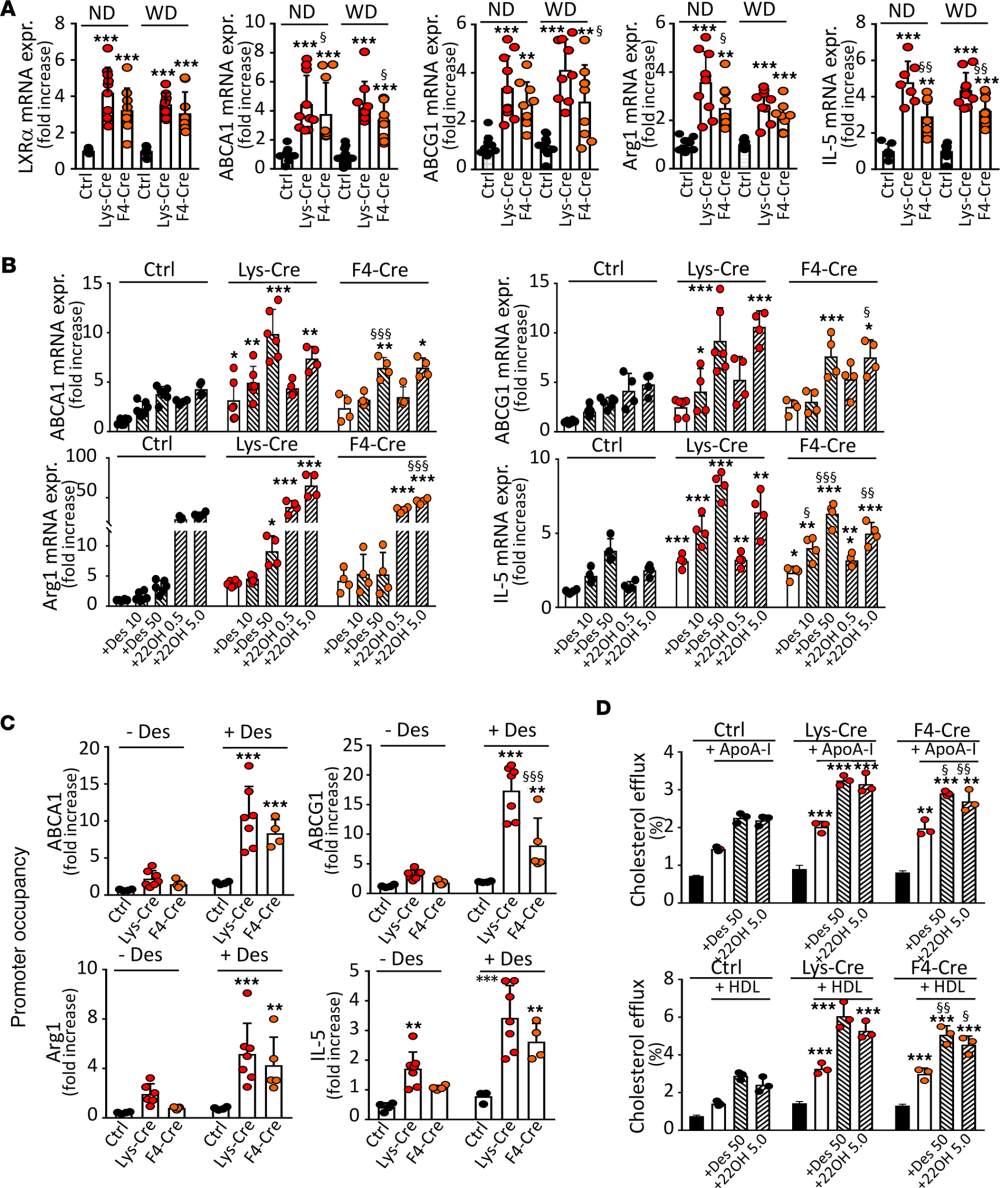
S1P_1_ overexpression in macrophages enhances expression and activation of LXRα. PMs from either *S1pr1-KI* (Ctrl, *n* = 7–10), *S1pr1-LysMCre* (Lys-Cre, *n* = 7–10), or *S1pr1-F4/80Cre* (F4-Cre, *n* = 7–10) mice on ND or *Ldlr^–/–^* mice transplanted with *S1pr1-KI* (*n* = 10), *S1pr1-LysMCre* (*n* = 9), or *S1pr1-F4/80Cre* (*n* = 9) BM on WD. (**A**) qPCR of *Lxra* and LXR signature genes. mRNA normalized to *Gapdh* and presented relative to *S1pr1-KI*. (**B**) PMs from ND-fed mice incubated for 24 hours in media with desmosterol (Des, 10 or 50 μmol/L) or 22-hydroxycholesterol/9-cis-retinoic acid (22OH, 0.5 and 5.0 μg/mL). qPCR of LXR signature genes (*n* = 4–6 for each group). (**C**) LXR occupancy at *Abca1*, *Abcg1*, *Arg1*, and *Il5* promoters by ChIP in PMs from ND-fed mice incubated for 24 hours with or without desmosterol (50 μmol/L). Primers amplifying at –85 bb and –4.0 kb for *Abca1*, –0.1 kb and –3.2 kb for *Abcg1*, –0.7 kb and –1.6 kb for *Arg1*, and –0.2 kb and –1.0 kb for *Il5* used as positive binding sites and negative controls (*n* = 4–7 for each group). (**D**) PMs from ND-fed mice loaded with [1,2-^3^H]-cholesterol (*n* = 3 for each group) were incubated for 4 hours with apoA-I (10.0 μg/mL) or HDL (12.5 μg/mL). * - *P* < 0.05, ** - *P* < 0.01, *** - *P* < 0.001 (Lys-Cre vs. Ctrl or F4-Cre vs. Ctrl), ^§^ - *P* < 0.05, ^§§^ - *P* < 0.01, ^§§§^ - *P* < 0.001 (Lys-Cre vs. F4-Cre, 1-way or 2-way ANOVA except **A** Lxra/WD and **A** Abca1/ND: Kruskal-Wallis *h* test).

In addition to IRF8- and PU.1-controlled genes, *S1pr1-LysMCre* PMs showed an increased expression of the liver X receptor–dependent (LXR-dependent) genes *Apoe* and *Pltp* (Figure 2A). Therefore, we investigated the effect of S1P_1_ overexpression on LXR activity. We found elevated mRNA expression of *Lxr* and its target genes ATP-binding cassette transporters A1 (*Abca1*) and G1 (*Abcg1*), arginase 1 (*Arg1*), and interleukin-5 (*Il5*) in *S1pr1-LysMCre* and *S1pr1-F4/80Cre* PMs (Figure 4A). Furthermore, the excessive mRNA expression of these genes was upregulated by LXR ligands desmosterol or 22-hydroxycholesterol with retinoic acid (22OH/RA, Figure 4B). In addition, the cell surface expression of LXR targets CD226 and CD244 was upregulated in *S1pr1-LysMCre* and *S1pr1-F4/80Cre* PMs (Supplemental Figure 4A). To assess LXR activity, we evaluated its binding to target gene promoters by ChIP. LXR promoter occupancy at –85 bp, –0.1 kb, –0.7 kb, and –0.2 kb binding sites in *Abca1*, *Abcg1*, *Arg1*, and *Il5*, respectively, was enhanced in macrophages from *S1pr1-LysMCre* and *S1pr1-F4/80Cre* mice and augmented by desmosterol (Figure 4C). Because of the elevated LXR activity, S1P_1_-overexpressing macrophages displayed increased cholesterol efflux to apoA-I or HDL, respectively (Figure 4D). In addition, these cells secreted more IL-5 in response to desmosterol or 22OH/RA, and elevated IL-5 was found in the plasma from *S1pr1-LysMCre* and *S1pr1-F4/80Cre* mice (Supplemental Figure 4B).

### S1P_1_ overexpression in macrophages inhibits apoptosis and promotes efferocytosis.

**Figure 5:**
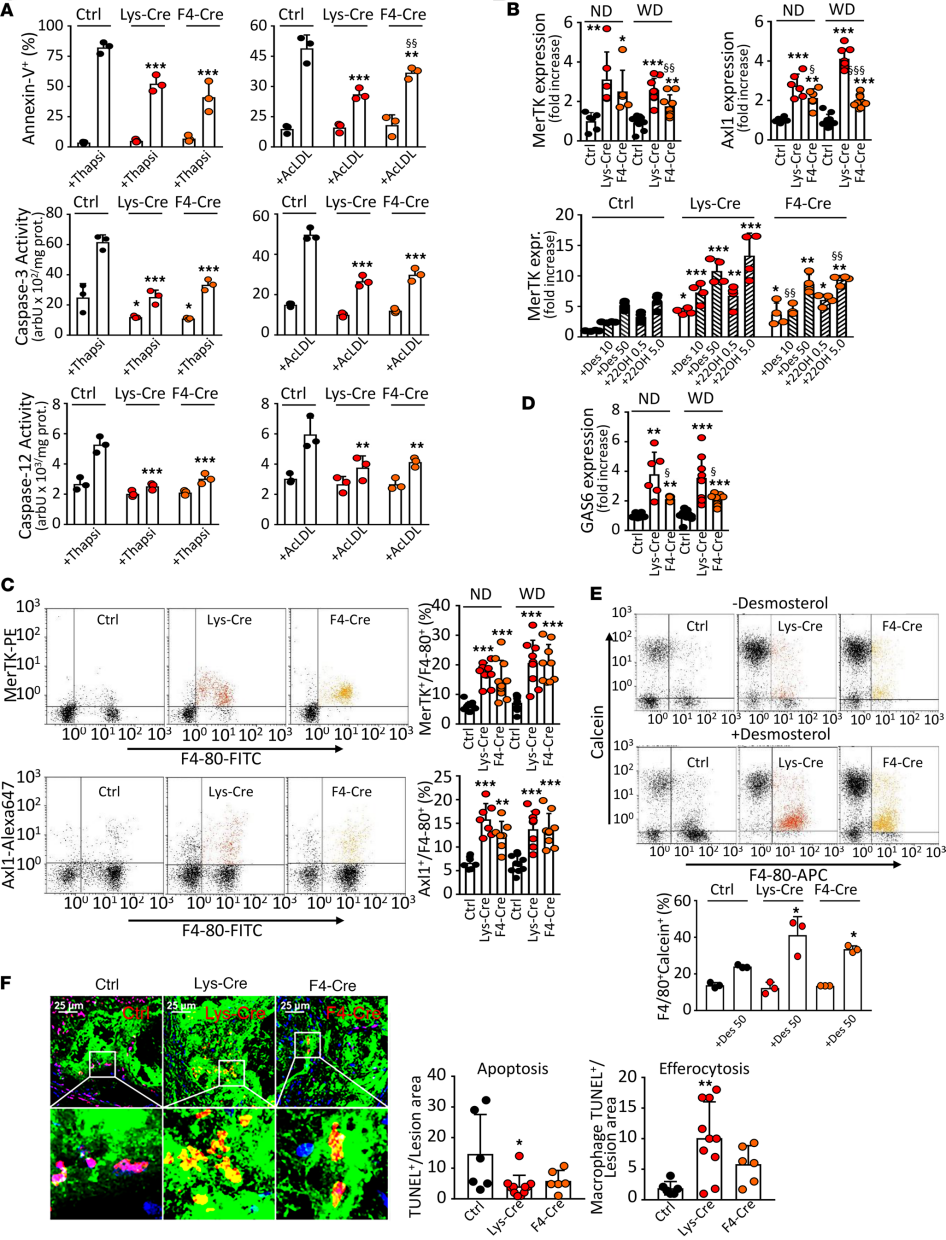
S1P_1_ overexpression in macrophages inhibits ER stress–dependent apoptosis and enhances efferocytosis. PMs from *S1pr1-KI* (Ctrl, *n* = 6–10), *S1pr1-LysMCre* (Lys-Cre, *n* = 6–10), or *S1pr1-F4/80Cre* (F4-Cre, *n* = 6–10) mice on ND or *Ldlr^–/–^* mice transplanted with *S1pr1-KI* (*n* = 10–11), *S1pr1-LysMCre* (*n* = 9), or *S1pr1-F4/80Cre* (*n* = 9) BM on WD. (**A**) PMs from ND-fed mice exposed for 24 hours to thapsigargin/fucoidan (Thapsi, 0.5 μmol/L and 25.0 μg/mL) or acetylated LDL (AcLDL, 100.0 μg/mL). Percentage of apoptotic (annexin V positive) cells and caspase-3 and -12 activities (*n* = 3 for each group). (**B**) qPCR of *Mertk* and *Axl1* mRNA normalized to *Gapdh* and presented relative to *S1pr1-KI*. Lower panel: *Mertk* in PMs from ND-fed mice incubated for 24 hours with desmosterol (10 or 50 μmol/L) or 22-hydroxycholesterol/9-cis-retinoic acid (0.5 and 5.0 μg/mL, *n* = 4 for each group). (**C**) MerTK and Axl1 analyzed by flow cytometry. (**D**) qPCR of *Gas6*. (**E**) Dot plots showing efferocytosis of apoptotic RAW264.7 cells (ATCC) by PMs from ND-fed mice incubated for 24 hours with or without desmosterol (50 μmol/L). RAW264.7 cells and PMs were labeled with calcein and anti–F4/80-FITC (*n* = 3 for each group). (**F**) Aortic root section images with apoptotic cells labeled by TUNEL (red), macrophages by anti–MOMA-2 (green), and nuclei by DAPI (blue). Apoptotic cells appear violet (red on blue), and efferocytotic cells appear yellow (red on green, *n* = 5–10 for each group). The side of the square inset box measures 36 µm. * - *P* < 0.05, ** - *P* < 0.01, *** - *P* < 0.001 (Lys-Cre vs. Ctrl or F4-Cre vs. Ctrl), ^§^ - *P* < 0.05, ^§§^ - *P* < 0.01, ^§§§^ - *P* < 0.001 (Lys-Cre vs. F4-Cre, 1-way or 2-way ANOVA except **B** Axl1/WD, **D** ND and WD, and **F** Apoptosis: Kruskal-Wallis *h* test).

Since the mRNA expression of antiapoptotic genes *Mafb* and *Bcl6* was increased in S1P_1_-overexpressing macrophages (Figure 2D), we examined the macrophage propensity to undergo ER stress–induced apoptosis. The transmembrane phosphatidylserine shift and caspase-3 activity were determined in PMs after ER stress induction with thapsigargin/fucoidan or cholesterol loading. In addition, the ER stress–induced caspase-12 activity was determined. All indicators were attenuated in *S1pr1-LysMCre* and *S1pr1-F4/80Cre* mice (Figure 5A). Moreover, the tendency toward lower caspase-3 activity in S1P_1_-overexpressing macrophages was reversed by the BCL6 inhibitor 79-6 or the MafB modulator bortezomib (Supplemental Figure 5). As the reduced apoptosis is coupled to more effective efferocytosis, we subsequently examined whether efferocytosis is altered in S1P_1_-overexpressing macrophages. We found that the mRNA expression of *Mertk* and *Axl1* — receptors responsible for the apoptotic cell ingestion and controlled by LXR and MafB, respectively (27, 28), was elevated in *S1pr1-LysMCre* and *S1pr1-F4/80Cre* PMs (Figure 5, B and C). In addition, both desmosterol and 22OH/RA augmented the mRNA expression of the LXR target *Mertk* in these cells (Figure 5B). Likewise, S1P_1_ overexpression enhanced the mRNA expression of growth arrest-specific 6 (*Gas6*), the bridging molecule between MerTK and apoptotic cells (Figure 5D). Consequently, desmosterol enhanced efferocytosis in S1P_1_-overexpressing macrophages as inferred from the increased ingestion of apoptotic RAW264.7 cells (Figure 5E). In addition, S1P_1_ overexpression affected apoptosis and efferocytosis in atherosclerotic lesions. We detected fewer macrophages with TUNEL-positive own nuclei but more macrophages with TUNEL-positive ingested nuclei within aortic root lesions, pointing to attenuated apoptosis but more efficient efferocytosis in *S1pr1-LysMCre* and *S1pr1-F4/80Cre* mice (Figure 5F).

### S1P_1_ agonist KRP203 emulates the effect of S1P_1_ overexpression on the functional phenotype in macrophages.

To strengthen the evidence underscoring the S1P_1_ effect on macrophage polarization, we treated both wild-type (WT) and *Ldlr^–/–^* mice on atherogenic diet with KRP203 — an S1P_1_ agonist with antiatherogenic properties (16) — and assessed their peritoneal macrophage phenotype. KRP203 substantially reduced absolute peripheral leukocyte and relative lymphocyte count in both mouse strains, verifying the treatment efficacy, but had no effect on body weight and plasma lipids in *Ldlr^–/–^* mice (Supplemental Figure 6, A and B). As shown in Supplemental Figure 6, C and D, changes in the mRNA expression of PU.1/IRF8-dependent genes (*Bcl6*, *Klf4*, *Mhc2ta*, and *Cd115*), the cell surface expression of polarization markers (CD206 and CD86), and the mRNA expression of LXR-dependent genes (*Abca1*, *Abcg1*, *Mertk*, and *Cd244*) in PMs from both WT and *Ldlr^–/–^* mice treated with KRP203 fully recapitulated the expression patterns seen in S1P_1_-overexpressing macrophages. In addition, the mRNA expression of *Cd68* was reduced in PMs from mice administered KRP203 (Supplemental Figure 6D). A similar pattern was exhibited in PMs exposed in vitro to the active S1P_1_ ligand KRP203-phosphate (1.0 μmol/L) for 24 hours and was further enhanced by the concomitant overexpression of S1P_1_ (Supplemental Figure 6E).

We were concerned that the Cre transgene might affect the animal phenotype, translating into some beneficial changes in PM function in S1P_1_-overexpressing hematopoietic chimeras. However, we observed no differences between WT (C57BL/6), *S1pr1-KI*, and *LysM-Cre* mice with respect to the functional macrophage phenotype (Supplemental Figure 7).

### S1P_1_ overexpression attenuates atherosclerosis and produces an antiatherogenic macrophage phenotype in Apom^–/–^ Ldlr^–/–^ mice.

To investigate whether binding to HDL is a prerequisite for S1P to unfold antiatherogenic activity, we employed *Apom^–/–^* *Ldlr^–/–^* mice, which lack the S1P chaperone apoM. As shown in Supplemental Figure 8, apoM deficiency did not abolish the favorable effect of S1P_1_ overexpression on the lesion area or macrophage phenotype (Supplemental Figure 8, A–D).

### S1P_1_ signals in macrophages via PKA and AKT.

**Figure 6:**
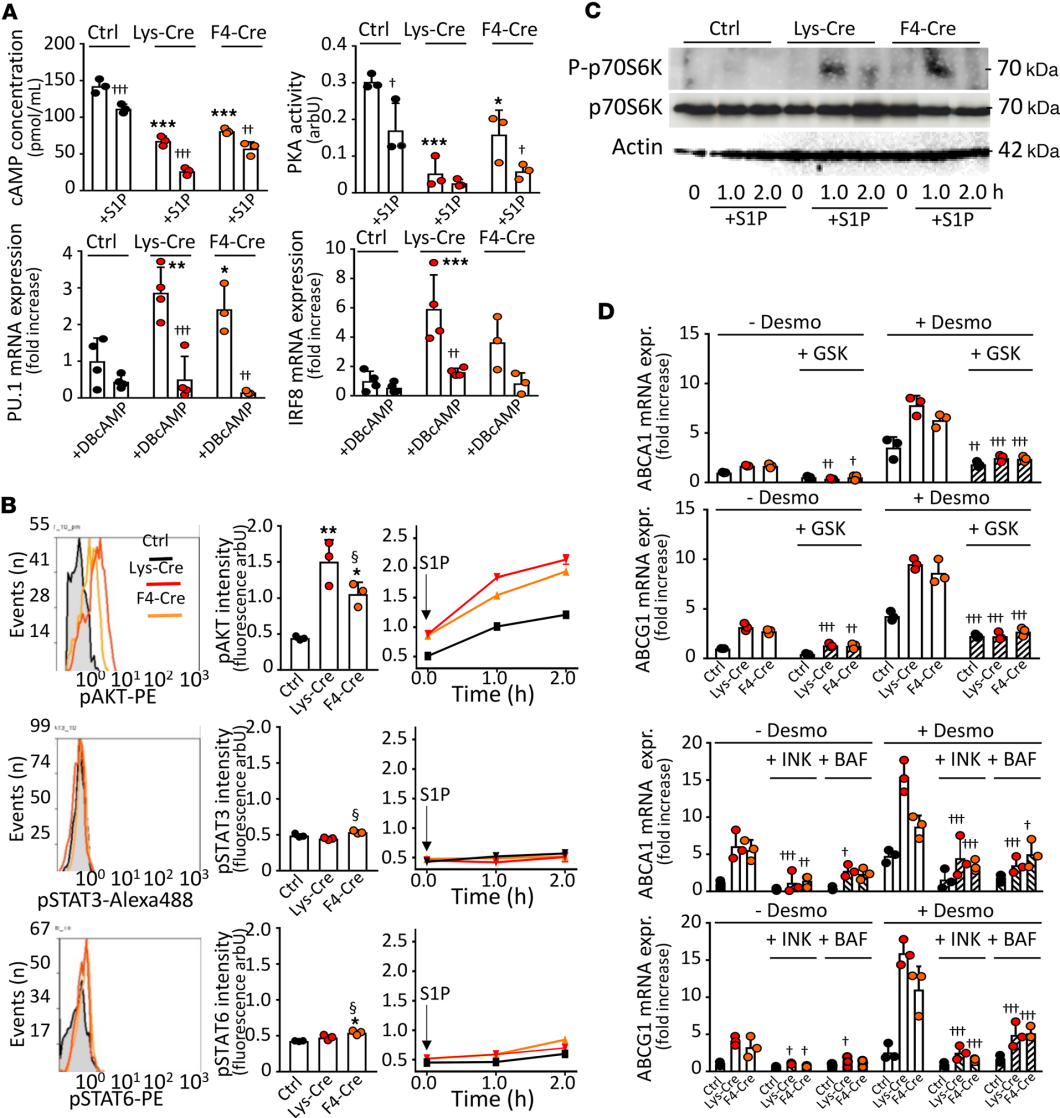
Stimulatory effects of S1P_1_ overexpression are mediated by PKA and AKT. PMs from *S1pr1-KI* (Ctrl, *n* = 3–4), *S1pr1-LysMCre* (Lys-Cre, *n* = 3–4), or *S1pr1-F4/80Cre* (F4-Cre, *n* = 3–4) mice on ND were established in culture. (**A**) Cells were exposed to S1P (1.0 μmol/L) for 2 hours (upper panels) or dibutyryl-cAMP (DBcAMP) (0.25 mmol/L) for 24 hours (lower panels). cAMP levels and PKA activity were measured using enzyme immunoassay or Pep-Tag assay. *Pu1* and *Irf8* expressions were analyzed by qPCR. (**B** and **C**) PMs were analyzed for kinase activities or exposed to S1P (1.0 μmol/L) for indicated times. Intracellular stainings for phospho-AKT, phospho-STAT3, and phospho-STAT6 analyzed by flow cytometry (**B**). For mTOR1 activity, PMs lysates probed with antibodies against total and phosphorylated (P) p70S6 kinase (**C**). Blots representative for 2 independent experiments. (**D**) Cells were exposed for 30 minutes to GSK690693 (10.0 μmol/L), INK128 (0.2 μmol/L), or bafilomycin (1.0 μmol/L) prior to incubation with desmosterol (50 μmol/L) for 24 hours. *Abca1* and *Abcg1* genes analyzed by qPCR. Shown are results from 3 independent experiments. ^†^ - *P* < 0.05, ^††^ - *P* < 0.01, ^†††^ - *P* < 0.001 with vs. without treatment with activator/inhibitor, * - *P* < 0.05, ** - *P* < 0.01, *** - *P* < 0.001 (Lys-Cre vs. Ctrl or F4-Cre vs. Ctrl), ^§^ - *P* < 0.05 (Lys-Cre vs. F4-Cre, 1-way or 2-way ANOVA).

Finally, we investigated signaling pathways accounting for the antiatherogenic phenotype in S1P_1_-overexpressing macrophages. Since PU.1 and LXR activities are inversely and directly regulated by PKA as well as AKT and mechanistic target of rapamycin complex 1 (mTORC1) (29–31), respectively, we examined the effect of S1P on these kinases. Both the PKA activity and concentration of its upstream regulator cAMP were lower in *S1pr1-LysMCre* and *S1pr1-F4/80Cre* PMs, and this was potentiated by exogenous S1P (Figure 6A). By contrast, basal and S1P-stimulated AKT activities were increased in S1P_1_-overexpressing macrophages (Figure 6B). Similarly, S1P treatment stimulated mTORC1 in *S1pr1-LysMCre* and *S1pr1-F4/80Cre* PMs, as indicated by the p70S6 kinase phosphorylation (Figure 6C). In addition, phosphorylation of the eukaryotic translation initiation factor 4E-binding protein 1 (4E-BP1) — a well-known mTOR substrate — was enhanced in *S1pr1-LysMCre* PMs (Supplemental Figure 9A). Preincubating PM with the PKA activator DBcAMP suppressed the elevated *Spi1* (PU.1) and *Irf8* mRNA expression (Figure 6A). Similarly, the elevated mRNA expression of LXR targets *Abca1* and *Abcg1* was abolished by inhibitors of AKT (GSK690693) and mTORC1 (INK128) (Figure 6D). In addition, *Abca1* and *Abcg1* mRNA expression was suppressed by bafilomycin (Figure 6D), which indirectly inhibits the late endosomal/lysosomal adaptor MAPK and mTOR activator 1 (Lamtor-1) — the scaffolding protein for mTORC1 activation and the element connecting the signaling between AKT, mTORC1, and LXR (31). The involvement of AKT was further verified using A-674563, a synthetic inhibitor of AKT1, which completely abolished desmosterol-mediated induction of *Abca1* and *Abcg1* mRNA expression in *S1pr1-LysMCre* and *S1pr1-KI* PMs, whereas CCT-128930, a specific inhibitor of AKT2, showed only weak inhibitory effects (Supplemental Figure 9B). In contrast with AKT, basal and S1P-stimulated activities of STAT3 and STAT6, which are involved in macrophage polarization (see Discussion), were unchanged in PMs from S1P_1_-overexpressing macrophages (Figure 6B).

### S1P_1_ overexpression exerts marginal effects on neutrophil plaque content and function in S1pr1-LysMCre mice.

In addition to macrophages, lysozyme M promoter–driven Cre also shows significant activity in neutrophils, which led to substantially increased *S1p1* mRNA and S1P_1_ cell surface expression in neutrophils isolated from the *S1pr1-LysMCre* BM as compared with the *S1pr1-KI* BM (Supplemental Figure 1C). Therefore, we investigated the effects of increased neutrophil S1P_1_ expression on neutrophil function and cell content in atherosclerotic lesions.

We observed a slight reduction in neutrophil content in aortic roots from *S1pr1-LysMCre*–transplanted *Ldlr^–/–^* mice (Supplemental Figure 10A). In addition, IL-10 and KC1/GROα production in response to lipopolysaccharide (LPS) was lower in *S1pr1-LysMCre* neutrophils (Supplemental Figure 10B). However, shedding of L selectin and ROS generation in response to LPS or PMA, both sensitive indicators of neutrophil function, were comparable in *S1pr1-LysMCre* and *S1pr1-KI* mice (Supplemental Figure 10, B–D).

## Discussion

Previous studies revealed that S1P signaling protects against atherosclerosis (16–20), but the identity of the S1P receptor subtype mediating this effect remained enigmatic. Studies employing S1P mimetics in mouse atherosclerosis models led to discrepant results, which was attributed to inadequate specificity or poorly defined side effects. Recently, accelerated lesion progression was reported in mice with endothelial or myeloid S1P_1_ deficiency (8, 20). However, the effect of cell-specific S1P_1_ upregulation on atherosclerosis was not examined. Here, we generated myeloid and macrophage-specific S1P_1_-knockin mouse lines with high and low S1P_1_ overexpression in macrophages, respectively, and used them as BM donors to produce *Ldlr^–/–^* chimeras prone to vascular lesion development. Analysis of atherosclerosis revealed reduced lesion areas in both lines, albeit the protective effects were more pronounced in *S1pr1-LysMCre* with high S1P_1_ overexpression than in *S1pr1-F4/80Cre* mice with low S1P_1_ overexpression. In addition, S1P_1_ overexpression blunted necrotic core formation, which critically depends on the macrophage apoptosis and removal of cellular debris within plaques. Accordingly, both *S1pr1-LysMCre* and *S1pr1-F4/80Cre* mice exhibited diminished apoptosis within atherosclerotic lesions along with an increased total and efferocytosis-positive macrophage number. These findings combined with the lower collagen amount in *S1pr1-LysMCre* lesions that could not be attributed to the increased expression of collagen-degrading proteases in macrophages point to slower advancement of lesions in S1P_1_-overexpressing chimeras. Collectively, these data for the first time to our knowledge provide unequivocal evidence that the amplification of S1P_1_-mediated signaling in macrophages protects against the development of atherosclerosis.

Although both the LysM and F4/80 promoters direct gene expression exclusively in the myeloid lineages, the 2 S1P_1_-overexpressing chimeras presented with quantitatively distinct phenotypes. While *S1pr1-LysMCre* mice showed a dramatic reduction of atherosclerosis combined with marked blood count alterations, the respective effects were moderate in *S1pr1-F4/80Cre* mice. Two explanations may account for this finding. First, considerably lower F4/80 than LysM promoter activity was predicted in silico in PMs, and this effect was even more pronounced in tissue-resident macrophages (see Supplemental Figure 1B). Accordingly, we found roughly 20%–50% weaker gene expressions, promoter occupancies, and/or functional cell responses in *S1pr1-F4/80Cre* as compared with *S1pr1-LysMCre* macrophages. These findings are congruent with previously published empirical results demonstrating that Cre expression from the F4/80 promoter is lower than from the LysM promoter in PMs and to an even greater extent in tissue-resident macrophages, which are primarily involved in the development of vascular lesions (32). Thus, the less pronounced antiatherogenic effect seen in *S1pr1-F4/80Cre* mice could be related to the quantitatively lesser enhancement of S1P_1_ signaling in macrophages in this mouse line. Second, our in silico analysis predicted marked LysM, but not F4/80, promoter activity in neutrophils in addition to macrophages. As neutrophils are now firmly identified as important players in the pathogenesis of atherosclerosis, the favorable antiatherogenic effect of S1P_1_ overexpression in these cells cannot be entirely dismissed. Of note, apoM-bound S1P has been recently shown to inhibit the formation of neutrophil extracellular traps and to increase the survival rate in a mouse model of LPS-induced sepsis (33). On the other hand, S1P_1_ seems to potentiate the neutrophil recruitment to chronically inflamed tissues, and inhibition of S1P_1_ signaling promotes the resolution rather than the exacerbation of neutrophilic inflammation (34, 35). With respect to atherosclerosis, little evidence could be found supporting the attenuating effect of S1P_1_ signaling in neutrophils on vascular lesion development in myeloid *S1pr1*-deficient mice (20). Our present results regarding the potential impact of neutrophil S1P_1_ on atherosclerosis remain inconclusive. While we observed a slight but significant reduction of neutrophil counts in aortas from *S1pr1-LysMCre*–transplanted mice, these cells were mainly localized in the adventitia rather than intima, making their impact on the atherosclerosis development questionable. In addition, while some proinflammatory neutrophil functions, such as cyto- and chemokine production, were reduced in cells obtained from *S1pr1-LysMCre*–transplanted mice, other important indicators of neutrophil activation (ROS production, L selectin shedding) were comparable to control cells. Notwithstanding this, BM transplantation from *S1pr1-F4/80Cre* mice with only moderate enhancement of S1P_1_ signaling exclusively in macrophages was sufficient to produce a measurable antiatherogenic effect in *Ldlr^–/–^* mice. This strongly supports the notion that the major antiatherogenic effect attributable to S1P_1_ takes place in macrophages and that potential antiatherogenic effects of S1P_1_ overexpression in neutrophils — if any — have only auxiliary character.

**Figure 7:**
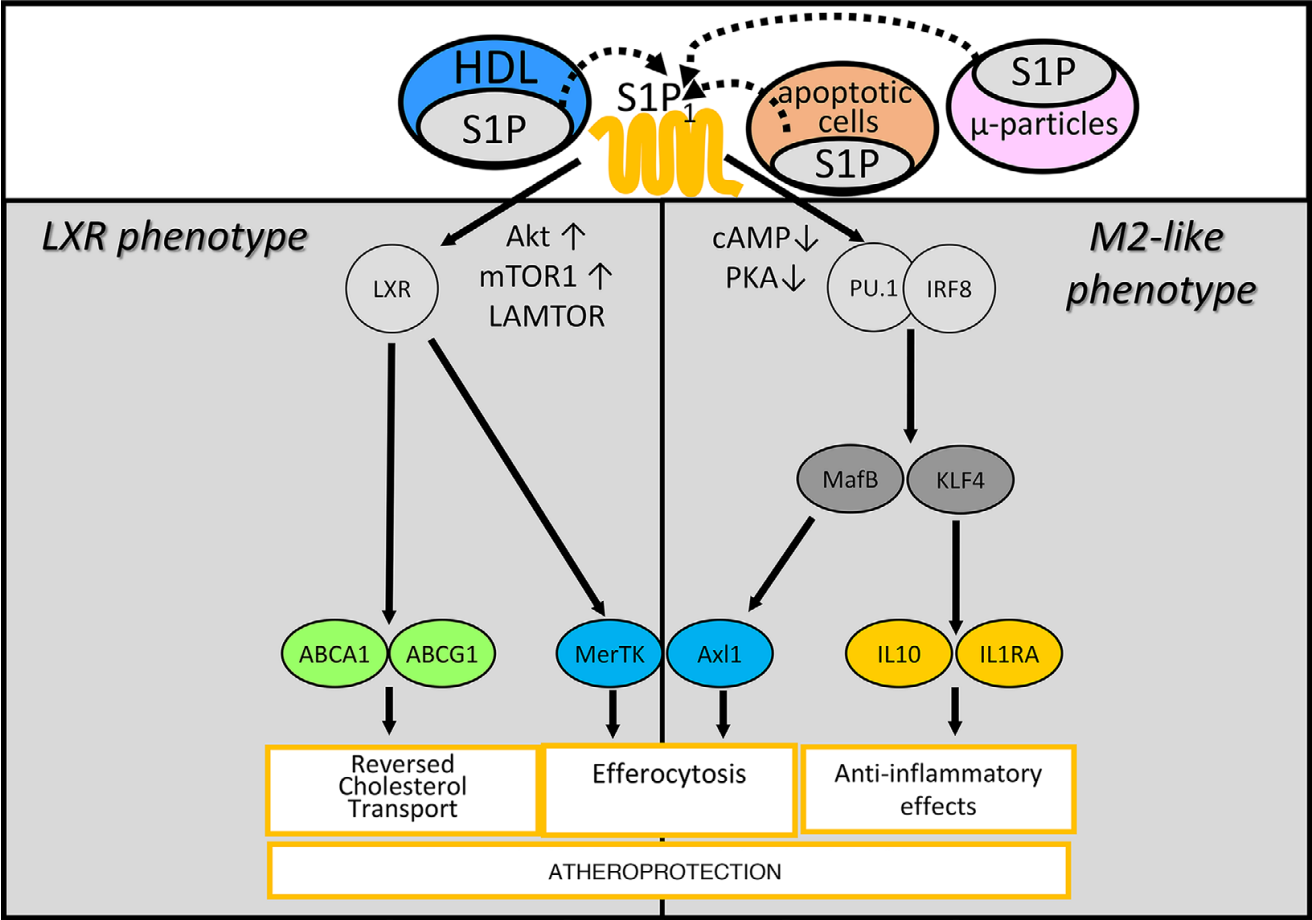
Proposed molecular mechanisms underlying atheroprotective effects of S1P_1_ signaling in macrophages. Two signaling pathways are triggered by S1P upon interaction with S1P_1_ in macrophages: First, lowering intracellular cAMP and PKA activity enhances the function of IRF8 and PU.1, which facilitates the development of an M2-like macrophage phenotype characterized by the increased production of antiinflammatory cytokines. Second, stimulation of AKT and mTOR1 fosters LXR activity and thereby promotes ABCA1– and G1–dependent reversed cholesterol transport. By elevating MerTK and Axl1 both pathways facilitate efferocytosis. The combined effect is the attenuation of the development of atherosclerotic lesions.

Our findings demonstrate a unique effect of S1P_1_ overexpression on the functional phenotype of macrophages. Previous studies indicated the role of S1P-induced signaling in promoting antiinflammatory macrophage polarization corresponding to the classical antiinflammatory M2 phenotype. For instance, the expression of pro-inflammatory genes (inducible nitric oxide synthase, cyclooxygenase) and the production of inflammatory cytokines were reduced in LPS-stimulated macrophages pretreated with S1P, while canonical markers defining the M2 phenotype (*Arg1*, *Ym1*, *Il10*, *Cd163*, *Cd206*) were upregulated (36–38). We here partially recapitulate these findings with respect to lower pro-inflammatory cyto- and chemokine production. Moreover, we extend them to define what we believe to be a novel, previously unrecognized functional phenotype distinct from the M2 polarization, which emerges both in S1P_1_-overexpressing macrophages and macrophages from mice treated with S1P_1_ agonist KRP203 as a result of the concomitant PU.1/IRF8 and LXRα activation. Previous studies documented that PU.1, acting in concert with transcription factors, such as IRF8, IRF4, STAT6, or HOXA3, promotes the expression of M2 polarization markers (*Arg1*, *Ym1*, *Fizz/Retnla*) and drives macrophages toward a corresponding phenotype distinguished by the increased production of MHC-II, CD206, IL-1RA, and IL-4 (23, 39–42). In addition, PU.1 controls transcription factors KLF4 and MafB, which support the sustained M2 macrophage polarization (25, 26). In our experimental setting, macrophages overexpressing S1P_1_ or treated with KRP203 acquired similar phenotypic features. However, reprogramming of macrophages toward the classical M2 phenotype was repeatedly shown to downregulate rather than upregulate LXRα-dependent gene expression (43, 44). Accordingly, macrophages generated by exposure to IL-4 displayed diminished, not increased, cholesterol efflux capacity owing to the reduced expression of *Lxra* and *Abca1* (44). In a remarkable contrast, both S1P_1_ overexpression and KRP203 treatment led to activation of LXRα, which entailed the upregulation of *Abca1*, *Abcg1*, *Il5*, and *Mertk*. Overall, the concomitant activation PU.1/IRF8 and LXRα generated a unique macrophage phenotype not identical with the M2 one, bundling several antiatherogenic mechanisms (Figure 7). First, due to enhanced expression of PU.1, KLF4, MafB, and LXRα, these cells acquired antiinflammatory properties, including the increased secretion of IL-10, IL-1RA, and IL-5, aiding to resolve inflammation and protect against atherosclerosis. Second, owing to the augmented LXRα activity, S1P_1_-overexpressing macrophages upregulated cholesterol transporters *Abca1* and *Abcg1* and cholesterol efflux capacity, which likely reduced plaque cholesterol burden. Third, the increased expression of *Bcl6* and *Mafb* attenuated the macrophage propensity to undergo ER stress–induced apoptosis, which translated into enhanced macrophage survival within atherosclerotic lesions. Fourth, due to the LXR- and MafB-dependent enhancement of *Mertk* and *Axl1* expression, these macrophages could also be expected to effectively efferocytize apoptotic cells, a process governing necrotic core formation and late-stage lesion progression. Collectively, the present findings support the contention that S1P_1_ acts as master regulator of a unique functional macrophage phenotype unifying multiple atheroprotective mechanisms (Figure 7). The emergence of such a phenotype in *S1pr1-LysMCre* and to a lesser extent in *S1pr1-F4/80Cre* mice provides a consistent explanation for the attenuated plaque formation in these animals.

Our findings provide additional mechanistic insights into signaling pathways linking S1P_1_ with the molecular machinery involved in the development of the atheroprotective phenotype in macrophages (Figure 7). By coupling to the trimeric G_i_ protein, S1P_1_ initiates signaling cascades, which in addition to the G protein class-defining inhibition of adenylate cyclase includes the activation of AKT (1–3). Accordingly, reduced cAMP concentration and PKA activity as well as increased AKT activity were seen in macrophages from *S1pr1-LysMCre* and *S1pr1-F4/80Cre* mice. By contrast, STAT3, which is activated by S1P_1_ in several tumor cell lines (45), remained quiescent in S1P_1_-overexpressing macrophages. Likewise, S1P_1_ overexpression did not affect STAT6, which participates in the development of the M2 phenotype in macrophages exposed to IL-4 (46). Previous studies reported decreased PU.1 promoter binding in macrophages upon elevated intracellular cAMP and PKA activation (29, 30), which is congruent with the reversal of increased PU.1/IRF8 expression in S1P_1_-overexpressing macrophages exposed to the cAMP mimetic DBcAMP. We assume that the diminished cAMP content coupled to the enhanced PU.1 and IRF8 expression in these cells translated into the antiinflammatory macrophage phenotype and reduced atherosclerosis in *S1pr1-LysMCre* and *S1pr1-F4/80Cre* mice. Moreover, previous reports associated AKT activation in macrophages with diverse antiatherogenic effects. For instance, AKT promotes macrophage survival by inhibiting caspase-3 or stimulating the transcriptional activation of antiapoptotic genes (47). Moreover, impaired efferocytosis was observed in macrophages obtained from LDL-related protein 1–deficient mice characterized by a defective AKT activation (47, 48). Finally, upregulation of AKT activity promotes antiinflammatory macrophage polarization (48). Out of 3 AKT isoforms expressed in macrophages, only AKT1 was unequivocally identified as atheroprotective, whereas AKT2 seems to exert some pro-atherogenic activity, as its loss in hematopoietic cells reduces plaque development in *Ldlr^–/–^* mice (47, 49). These findings are in line with our study, which shows that the potentially antiatherogenic signaling cascade triggered by S1P_1_ activation can be primarily linked to AKT1. Of note, AKT was identified as a triggering component of the signaling cascade encompassing mTORC1, the lysosomal adaptor Lamtor-1, and LXR, culminating in the M2 polarization of macrophages (31). In line with these findings, we observed that LXR activation in S1P_1_-overexpressing macrophages was dependent on AKT, mTORC1, and Lamtor-1, as it was abolished by respective inhibitors. Collectively, our findings point to AKT — most likely AKT1 — as an important mediator of the antiatherogenic effect exerted by S1P_1_ in macrophages.

Previous studies revealed that only apoM^+^ HDL-bound and not albumin-bound S1P augments the barrier function and inhibits leukocyte adhesion and apoptosis in endothelial cells via S1P_1_ (8, 11, 12). However, in our study, the transplantation of *Ldlr^–/–^* mice with S1P_1_-overexpressing BM reduced atherosclerosis and promoted antiatherogenic macrophage phenotype regardless of apoM expression. This finding does not support the contention that S1P mediates antiatherogenic effects of HDL in macrophages, though it may be required for the atheroprotective action in endothelial cells. Alternatively, other S1P chaperones in HDL might account for its antiatherogenic action in the absence of apoM. In this context, it is of interest that apoA-IV, which is a constituent of an HDL subfraction, was recently demonstrated to bind and present S1P to its receptors (50). In addition, Pltp was proposed to act as an S1P binding protein in plasma (51). Finally, it cannot be excluded that atheroprotective effects of non-HDL-bound S1P, which is present in atherosclerotic lesions as a component of apoptotic bodies or endothelially derived microparticles, becomes particularly evident under conditions of S1P_1_ overexpression. Clearly, further studies in alternative animal models are necessary to delineate the contribution of S1P to the antiatherogenic potential of HDL.

In conclusion, our study documents that the amplification of S1P_1_-dependent signaling in monocytes and macrophages countervails the lesion development in a mouse model of atherosclerosis. The underlying molecular mechanism involves the emergence of what we believe to be a novel macrophage phenotype, in which the parallel activation of transcription factors PU.1/IRF8 and LXR orchestrates several antiatherogenic pathways, including enhanced secretion of antiinflammatory cytokines, cholesterol disposal and efferocytosis, as well as reduced ER stress–induced apoptosis. Further investigations will be required to understand whether the atheroprotective mechanisms of S1P identified here may contribute to the beneficial effect of this lysosphingolipid on cardiovascular risk inferred from observational studies in humans.

## Methods

### Sex as a biological variable.

Our study examined female mice because larger and more pronounced atherosclerotic lesions in female compared with male *Ldlr*^–*/*–^ mice are expected on a C57BL/6J background (52).

### Animals.

*C57Bl/6J-Gt(ROSA)26Sor^tm1(S1pr1)Geno^* mice (referred to as *S1pr1-KI*) were generated by Genoway by knocking in the floxed mouse *S1pr1* transgene into embryonic stem cells as described (53). Briefly, the mouse *S1pr1* cDNA controlled by the CAG promoter was engineered to contain a neomycin-stop cassette between promoter and the cDNA. Hence, the *S1pr1* cDNA was only expressed following its Cre-mediated removal. The construct was introduced into the Rosa 26 locus. For S1P_1_ overexpression in monocytic cells, *S1pr1-KI* were crossed to B6.129P2-*Lyz2^tm1(cre)Ifo^*/J mice (Jackson Laboratory; crosses referred to as *S1pr1-LysMCre*) or B6.129P2-*Adgre1^tm1(cre)Kpf^* mice (from K. Pfeffer, University of Düsseldorf, Düsseldorf, Germany; crosses referred to as *S1pr1-F4/80Cre*). Female B6.129S7-*Ldlr^tm1Her^/*J mice (Jackson Laboratory, referred to as *Ldlr^–/–^*, 6 to 8 weeks of age) or *Ldlr^–/–^* crossed to apoM-lacking Apom^tm1Cchr^ mice (provided in-house, referred to as *Apom^–/–^* *Ldlr^–/–^*, 6 to 8 weeks of age) underwent BM aplasia by irradiation (11 Gy) before the transplantation with *S1pr1-KI*, *S1pr1-LysMCre*, or *S1pr1-F4/80Cre* BM. Thereafter, animals were put on a WD (0.5% cholesterol, 21% fat; Altromin) for 14 weeks. *C57Bl/6J* WT mice (Charles River Laboratories) received i.p. injections of 0.075 mg KRP203 (Novartis) twice weekly for 4 weeks. For euthanasia, mice were anesthetized with 5% (v/v) isoflurane introduced via a vaporizer followed by exsanguination by heart puncture.

### Materials and analytical procedures.

The detailed description of analytical procedures and materials used can be found in the online supplement (Supplemental Methods and Supplemental Tables 3–5).

### Statistics.

Data are presented as means ± SD from at least 3 independent determinations. The distribution normality was assessed either with Smirnov-Kolmogorov or with Shapiro-Wilk tests. Comparisons between 2 groups were performed with Student’s 2-tailed *t* test or Mann-Whitney test for normally and non-normally distributed populations, respectively. Comparisons between 3 or more groups were performed with 1- or 2-way ANOVA with Holm-Šidák test for pairwise post hoc comparisons or Kruskal-Wallis *h* test with Conover test for pairwise post hoc comparisons for normally and non-normally distributed populations, respectively. *P* values less than 0.05 were considered significant.

### Study approval.

All experiments conformed to the guidelines from directive 2010/63/EU and were approved by the local animal protection authorities (LANUV, Recklinghausen, Germany, permissions 84-02.04.2015.A505 and 81-02.04.2022.A329).

### Data availability.

Values for all data points in graphs are reported in the Supporting Data Values.xls file. The microarray data have been deposited in the ArrayExpress database at EMBL-EBI (www.ebi.ac.uk/arrayexpress) under accession number E-MTAB-14469. Other data from this study are available upon reasonable request.

## Author contributions

FP, RB, and JRN initiated and designed the study; FP, ES, RF, JF, LA, GV, EP, MDS, GL, BH, MF, and AHL performed experiments; FP, GV, RB, and JRN performed the statistical analyses, interpreted the results, and drafted the manuscript; AHL, JR, BEK, FR, TR, CC, and MS helped interpret the results, critically read the manuscript, and provided important resources and methodological aid. FP, MS, RB, MF, and JRN acquired funding.

## Supplementary Material

Supplemental data

Unedited blot and gel images

Supporting data values

## Version 1 11/12/2024

In-Press Preview

## Version 2 12/20/2024

Electronic publication

## Version 3 12/26/2024

Text spacing ommision in title: the space between “1

## Version 4 01/28/2025

Figures 2, 3, and 5 had parts of the image shifted. They now appear correctly
