# Supplementary material for: Sphingosine 1-phosphate receptor 1 signaling in macrophages reduces atherosclerosis in LDL receptor–deficient mice
Source: JCI Insight. 2024 Nov 12;9(24):e158127. doi: 10.1172/jci.insight.158127 (PMC11665566; doi:10.1172/jci.insight.158127)
Supplement: Supplemental data [file jciinsight-9-158127-s008.pdf]

Poti et al.: Sphingosine-1-Phosphate (S1P) receptor type 1 signaling in macrophages reduces atherosclerosis in LDL receptor-deficient mice

SUPPLEMENTAL MATERIALS

METHODS

Reagents – Compound 79-6 and bortezomib were obtained from Selleck Chemicals (München, Germany). GSK690693 and bafilomycin were from Tocris Bioscience (Bristol, GB). Cell culture reagents including DMEM, RPMI1640, non-essential amino acids and sodium pyruvate were from Lonza (Wuppertal, Germany). KRP-203 (2-amino-2-{3-[4-(3-benzyloxy-phenylsulfanyl)-2-chloro-phenyl]-ethyl}-propan-1,3-diol hydrochloride) and KRP-203-phosphate were provided by Novartis (Basel, Switzerland). Fetal calf serum (FCS) was from PAA Laboratories, Cölbe, Germany. Unless indicated otherwise, all other chemicals were from Sigma (Deisenhofen, Germany), and were of highest purity available.

Animals – *C57Bl/6J-Gt(ROSA)26Sor$^{tm1(S1pr1)Geno}$* mouse (referred to as *S1pr1-KI*) was generated by Genoway (Lyon, France) by knocking in the floxed murine *S1pr1 (S1p1)* transgene into ES cells. Briefly, the murine *S1p1* cDNA controlled by the synthetic CAG promoter was engineered to contain a neomycin-stop cassette between promoter and the cDNA. Hence, the *S1p1* cDNA is only expressed following Cre-mediated removal of the cassette. The construct was introduced into the Rosa 26 locus using the GenOway proprietary targeting vector. To achieve $S1P_1$ overexpression in monocytic cells, *S1pr1$^{f/stop/f}$* were crossed to B6.129P2-*Lyz2$^{tm1(cre)Ifo}$*/J mice (purchased from Jackson Laboratories, Bar Harbor, ME, [1]) or B6.129P2-*Adgre1$^{tm1(cre)Kpf}$* (gift of Klaus Pfeffer, University of Düsseldorf, Germany [2]) to yield *S1pr1$^{f/stop/f}$-Lyz2$^{tm1(cre)Ifo}$*/J mice (referred to as *S1pr1-LysMCre*) or *S1p1$^{f/stop/f}$-Adgre1$^{tm1(cre)Kpf}$* (referred to as *S1pr1-*

F4/80Cre). Genotyping was done by PCR using tail or ear biopsies. Animals were maintained in individually ventilated cages under a 12-hour light/12-hour dark cycle with free access to water and regular chow diet (66% carbohydrate, 12% fat, 22% protein).

Female *Ldlr*$^{-/-}$ mice on a C57BL/6J background (B6.129S7-*Ldlr*^{tm1Her}/J, [3], referred to as *Ldlr*$^{-/-}$) were purchased from Jackson Laboratories. *Ldlr*$^{-/-}$ crossed to apoM-lacking *Apom*^{tm1Cchr} mice backcrossed at least 7 times onto a C57B6/J background ([4] referred to as *Apom*$^{-/-}$/*Ldlr*$^{-/-}$, 6 to 8 week of age) were provided by C. Christoffersen. To induce bone marrow aplasia, *Ldlr*$^{-/-}$ mice (6-8 week of age) were exposed to a single total dose of 11 Gy total body irradiation 1 day before the transplantation. Bone marrow (BM) was isolated by flushing femurs and tibias from female *S1pr1-KI, S1pr1-LysMCre or S1p1-F4/80Cre* mice with phosphate-buffered saline (PBS) and single-cell suspensions were prepared by passing the cells through a 70 µm cell strainer. Irradiated recipients received 5.0 x 10^6 cells by intravenous injection into the tail vein. The hematological chimerism of transplanted animals was determined in genomic DNA from blood leukocytes 4 weeks after transplantation. Thereafter, animals were put on Western-type diet (0.5% cholesterol, 21% fat (corn oil, Research Diets D12107, manufactured by Altromin, Lage, Germany) for 14 weeks. At the end of the treatment period mice were sacrificed by exsanguination by heart puncture or cervical dislocation, both under anesthesia (5.0% isoflurane introduced via a vaporizer) and tissues were collected for further analysis.

C57BL/6J mice (referred to as wild type (WT)) were purchased from Charles River Laboratories (Sulzfeld, Germany). Mice (8 – 10 weeks, ca. 25 g, female) received twice a week i.p. injections of 0.075 mg KRP-203 (approx. 3.0 mg/kg/day) or the same volume (0.1 mL) of saline (control group). At the end of the treatment period mice were bled by heart puncture under complete anesthesia, peritoneal macrophages were

isolated and blood was collected for further analysis. All experiments conformed to the guidelines from directive 2010/63/EU and were approved by the local animal protection authorities (LANUV, Recklinghausen, Germany, permit Nr. 84-02.04.2015.A505 and 81-02.04.2022.A329).

Lipid and lipoprotein isolation, modification and determination – Low and high density lipoproteins (LDL and HDL) were isolated from the pooled plasma of healthy blood donors by a discontinuous potassium bromide gradient centrifugation (d=1.125-1.210 g/mL for LDL and d=1.125-1.210 g/mL for HDL). LDL was acetylated by the method of Fraenkel-Conrat [5] with minor modifications. Briefly, equal volumes of LDL and saturated sodium acetate were continuously mixed in ice-water bath, while glacial acetic acid (1.5 µL/1.0 mg total LDL protein) was added in 1,5 µL aliquots every 5 minutes. After addition acetic anhydrate mixture was stirred for additional 30 min and dialyzed for 24 h at 4°C against PBS (pH 7.4). Apolipoprotein A-I (ApoA-I) isolated from human plasma was obtained from Calbiochem, (Merck, Darmstadt, Germany). Mouse plasma lipoproteins were isolated by sequential ultracentrifugation from 60 µL of plasma at densities (d) of d<1.006 g/mL (VLDL), 1.006≤d≤1.063 g/mL (IDL, LDL) and d >1.063 g/mL (HDL) as described [6]. Cholesterol and triglyceride concentrations in plasma or isolated lipoprotein fractions were determined enzymatically using colorimetric assay kits (Roche Diagnostics, Mannheim, Germany). Plasma S1P levels were determined by liquid-chromatography tandem mass spectrometry as published previously [7].

Histology and Lesion Analysis - Atherosclerosis in the aortic root and brachiocephalic artery (BCA) was determined in a blinded fashion following the current recommendations of the AHA [8] and as previously described [9]. In brief, aortic roots and BCAs were removed under a dissecting microscope, embedded in O.C.T. (ThermoFisher Scientific, Schwerte, Germany) and snap-frozen at -80°C. Cross

sectional lesion areas at the aortic root were quantified in 5 oil red O-stained sections in the region beginning at the end of the aortic sinus and extending into the ascending aorta. Each of the five sections were separated by 50 µm. In addition, atherosclerotic lesions lumenal to the internal elastic lamina were quantified in 3 oil red O-stained sections per BCA. For both, aortic root and BCA, mean lesion sizes were calculated for each animal. Histological stainings were performed on single sections directly adjacent to oil red O-stained tissue sections. Macrophage content of lesions was determined by immunohistochemistry using specific antibodies against CD68 (AbD Serotec, Germany). Total collagen fiber content was analyzed in PicroSirius Red stained sections by polarization microscopy (Zeiss AxioObserver). Movat pentachrome staining to assess lesion quality was performed according to the manufacturer´s protocol (Morphisto, Germany). All lesions were photographed and analyzed using AxioVision KS400 image analysis (Carl Zeiss Microscopy GmbH, Germany).

Lesion apoptosis and efferocytosis quantitation - Apoptosis and efferocytosis in the aortic root cryosections were analyzed by confocal immunofluorescence microscopy. Serial 10 µm thick proximal aortic cryosections were stained with TUNEL (Tdt-Mediated dUTP Nick end Labeling) using the TMR red detection kit (Roche) according to the manufacturer instructions. Briefly, sections were fixed with 2% diluted neutral buffered formalin solution, permeabilized with a fresh prepared buffer containing Triton X-100 (0.1% v/v) in sodium citrate (0.1% w/v) and stained with TUNEL TMR red for 1 h at 37°C. Subsequently, sections were blocked with BSA solution (1.0 % v/v in PBS) and then incubated with anti-mouse MOMA-2 rat antibody (1:100 (v/v)), Santa Cruz Biotechnology, Heidelberg, Germany), overnight at 4°C. After washing, sections were incubated with goat anti rat IgG DyLight 488 (1:300 (v/v), Novus Biologicals, Centennial, CO) for 1 h at room temperature and protected from light. Slides were mounted with 4′,6-diamidino-2-phenylindole (DAPI) containing mounting media for

nuclear counterstaining. Images were acquired using the Leica TCS SP2 confocal microscope (Leica, Wetzlar, Germany), and then processed by at least two different operators using Fiji open-source image processing software [10]. The TUNEL positive nuclei were quantitated and normalized to the lesion area. In lesions where the TUNEL stain was condensed, fragmented, and/or faded, the average area of healthy DAPI-stained nuclei was used as reference to quantitate TUNEL positive signals. The free versus macrophage-associated TUNEL stain in the same sections were quantitated as described [11-13] and accordingly expressed. TUNEL positive nuclei were counted as free when they were not associated with or in close proximity to viable macrophages that were detected as clearly MOMA-2-stained macrophage cytoplasm surrounding a DAPI-stained nucleus.

Neutrophil staining of aortic root sections - Neutrophils were stained by a standard cytochemical method based on the Naphthol AS-D Chloroacetate Esterase (NACE) reaction, which is specific for cells of the granulocyte lineage and absent in monocytes or lymphocytes [14,15]. Cryosections of aortic root were stained according to the manufacturer's instructions (Sigma). Briefly, formalin fixed air-dried cryosections and all the reagents were warmed at 37°C. Sections were sequentially exposed to different staining solutions, buffers and rinsing steps. At the end, nuclei were counterstained with Mayer’s hematoxylin and glass slides mounted with an aqueous mounting media. Images were captured with a Nikon Optiphot 2 microscope, equipped with DS-U2 camera system, and analyzed with the Fiji (ImageJ) software [10] by two independent operators.

Leukocyte differential count and subtyping – Differential leukocyte count was performed on an automated hematology analyzer (XN1000, Sysmex Deutschland GmbH, Norderstedt, Germany) in a routine hospital laboratory. White blood cell subtyping was performed by flow cytometry. Briefly, whole blood was anti-coagulated

with citrate, incubated for 30 minutes with fluorescein isothiocyanate (FITC)-, phycoerythrin (PE)-, allophycocyanin (APC)- or allophycocyanin/cyanin 7 (APC/Cy7)-conjugated antibodies against CD11b, Ly6g, F4/80, CD193, CD3, CD4, CD8, B220 (each 5.0 µg/mL), and fixed for 30 minutes with 1% formaldehyde in phosphate buffered saline (PBS). Thereafter, cells were centrifuged for 10 minutes at 1700 rpm and erythrocytes were lysed in a buffer containing 0.15 mol/L NH_4Cl, 10 mmol/L $NaHCO_3$, 0.1 mmol/L EDTA (pH 7.4). The remaining cells were washed twice in PBS and analyzed on a CyFlow Space flow cytometer (Sysmex Partec, Münster, Germany) equipped with a 488 nm argon laser.

Isolation of macrophages and neutrophils - Peritoneal leukocytes were isolated by peritoneal lavage with ice-cold PBS as described previously [16,17]. Cells were suspended in DMEM containing FBS (10.0 % v/v) and 2 mmol/L glutamine and were either used for flow cytometry or seeded in a 12- 24- or 96-well cell culture plate at a required density. After 2 h non-adherent cells were removed, and remaining macrophages were harvested and analyzed or used for further experiments. For isolation of neutrophils, femurs and tibias harvested from sacrificed mice were flushed out with PBS without Ca^{2+}/Mg^{2+}. After lysis of erythrocytes the cell suspension was washed, centrifuged, and incubated in PBS on ice for 30 minutes. Subsequently, cells were resuspended in the MojoSort™ buffer (BioLegend, SanDiego, CA) adapted for cell separation using magnetic nanobeads. The cell count was adjusted to 1 x 10^8/ml and the neutrophil-negative magnetic sorting was carried out using MojoSort™ Mouse Neutrophil Isolation Kit (BioLegend) exactly according to the supplier protocol.

Analysis of gene expression by real-time quantitative RT-PCR - Total RNA was isolated from peritoneal macrophages using Trizol reagent (ThermoFisher Scientific) according to manufacturer protocol. RNA was eluted in water and quantified using BioPhotometer (Eppendorf, Hamburg, Germany). The entire cDNA was synthesized

from 1.0 µg of total RNA using RevertAid H Minus First Strand cDNA Synthesis Kit (ThermoFisher Scientific). PCR products were detected using ABI7900ht sequence detection system (Applied Biosystems, Darmstadt, Germany) in a 384-well format, using SYBR™ Green PCR master mix (ThermoFisher Scientific). PCR primer sequences are shown in Table S1. Relative gene expression was calculated by applying the $2^{-\Delta\Delta Ct}$ method.

Analysis of gene expression by microarray and pathway analysis - The peritoneal leukocytes were isolated as described above and seeded on a 24-well plate at density 4 x 10^5 cells/mL. After 2h non-adherent cells were removed and macrophages covered with RNeasy Lysis Buffer (RLT buffer, Qiagen, Venlo, The Netherlands). RNA was isolated using RNeasy Micro kit (Qiagen) according to the instruction of the manufacturer. The concentration and purity of RNA was analyzed on BioPhotometer. RNA integrity was assessed with the Agilent 2100 Bioanalyzer (Agilent Technologies Inc., Palo Alto, California). After cRNA labeling and hybridization to the Illumina HT-8 expression bead chips, microarrays were scanned on an iScan array scanner (Illumina, SanDiego, CA) and raw array data were processed and background-subtracted in Illumina GenomeStudio. Further analysis was performed using the Chipster open-source platform [18]. Expression values were quantile normalized and log2-transformed using the Bioconductor package ‘lumi’ implemented in Chipster. Statistical comparison between the sample groups was done within Chipster using the empirical Bayes method [19] and the Benjamini-Hochberg (BH) multiple-testing correction of the raw p-values. FDR threshold of 5% ($q < 0.05$) was used for filtering differentially expressed genes. The microarray data have been deposited in the ArrayExpress database at EMBL-EBI (www.ebi.ac.uk/arrayexpress) under accession number.

Pathway analysis to identify enriched pathways and processes was performed with the online software Metascape (www.metascape.org), using default parameters [20].

For genes with >2-fold upregulation in *S1pr1-LysMCre* mice, pathway and process enrichment analysis has been carried out with the following ontology sources: GO Biological Processes, KEGG Pathway, Reactome Gene Sets and CORUM. All genes in the genome have been used as the enrichment background. Terms with a p-value < 0.01, a minimum count of 3, and an enrichment factor > 1.5 (the enrichment factor is the ratio between the observed counts and the counts expected by chance) were collected and grouped into clusters based on their membership similarities. More specifically, p-values were calculated based on the accumulative hypergeometric distribution, and q-values were calculated using the Benjamini-Hochberg procedure to account for multiple testing.

Chromatin immunoprecipitation (ChIP) – Peritoneal leukocytes were seeded in 12-well plates at a density of 2 x 10^6 cells/mL and non-adherent cells were removed by washing with PBS. In some experiments, peritoneal macrophages were exposed overnight to desmosterol (50.0 µmol/L) in DMEM containing FBS (10.0 % (v/v)). Otherwise, the chromatin cross-linking was carried out directly. To this purpose, formaldehyde solution was added to the wells at the final concentration of 1.0 % (w/v) and incubated under shaking for 10 min. Cross-linking was subsequently quenched by the addition of glycine to a final concentration of 0.15 mol/L. The cells were rinsed twice, collected by scrapping in the ice-cold PBS and pelleted by centrifuging at 290g for 5 min at 4 °C. Afterwards, cells were washed 2 x in PBS and the nuclei fraction was collected by centrifugation (850 x g, 5 min., 4°C) and flash frozen.

Chromatin digestion was accomplished enzymatically using micrococcal nuclease (Cell Signaling Technology, Frankfurt, Germany). Briefly, the nuclear pellet was resuspended up in ice-cold SimpleChIP® enzymatic cell lysis buffer A supplemented with protease inhibitor cocktail (Cell Signaling Technology) and dithiothreitol (0.5 mmol/L), incubated for 10 minutes on ice and centrifuged (2000 x g, 5 minutes, 4°C).

Cells were washed in buffer B (1.0 mL), centrifuged again and resuspended in 0.1 mL of the cell lysis buffer B. The micrococcal nuclease (0.5 µL) was added for 20 minutes and the digestion was stopped using EDTA (0.5 mol/L). Thereafter, samples were spinned down and the pellet was resuspended in RIPA buffer (Tris-HCl 10 mmol/L pH 8.0, 1.0 mmol/L EDTA, 140 mmol/L NaCl, 1% (v/v) Triton X-100, 0.1% SDS (v/v), and 0.1% (w/v) Na-deoxycholate (w/v)) and sonicated on ice (3 x 20 sec).
Immunoprecipitation was performed overnight at 4°C using goat polyclonal antibody against IRF8 (ThermoFisher Scientific) and rabbit polyclonal antibodies against PU.1 and LXRα/β (both SantaCruz Biotechnology) coupled to Dynabeads protein G (ThermoFisher Scientific). Antibody coupling was performed exactly as described by Blecher-Gonen et al. [21]. Protein G-coupled antibodies were used in following amounts: IRF8 - 10.0 µg/reaction, PU.1 - 1:50 (v/v), LXRα/β - 3.0 µg/reaction. Rabbit or goat IgG (Cell Signaling Technology or SantaCruz Biotechnology) were included in the amount corresponding 1/10 of respective immunoprecipitating antibodies. Afterwards, the beads were washed with RIPA buffer (5 x), RIPA 500 buffer (Tris-HCl 10,0 mmol/L pH 8.0, 1.0 mmol/L EDTA, 500 mmol/L NaCl, 1% (v/v) Triton X-100, 0.1% SDS (v/v), and 0.1% (w/v) Na-deoxycholate (w/v), 2 x), LiCl wash buffer (10.0 mmol/L Tris-HCl pH 8.0, 1.0 mmol/L EDTA, 250 mmol/L LiCl, 0.5% (v/v) NP-40, 0.5% (v/v) Na-deoxycholate (w/v), 2 x), and TE buffer (10.0 mmol/L Tris-HCl pH 8.0, 1.0 mmol/L EDTA pH 8.0, 1 x) and finally resuspended in 50 µL of elution buffer containing 10 mmol/L Tris-HCl pH 8, 5 mmol/L EDTA, 300 mmol/L NaCl and 0.5% SDS (v/v). Next, 2 µL of RNAse (DNA-free, Roche) was added to each sample for 30 min at 37 °C followed by proteinase K (1.0 U/reaction, ThermoFisher Scientific) and glycogen (20 mg/reaction, Roche) were added to samples for further 2 h at 37°C. The incubation was continued overnight at 65°C to elute the DNA and reverse the cross-link. DNA was purified from supernatants using SPRI magnetic beads (Applied Biological Material,

Richmond, Canada) added in a 2.3 x ratio (v/v) for 2 min. The beads were washed twice with ethanol (70% (v/v)) and DNA was eluted with water and analyzed in real time PCR. Calculated results are normalized to input sample of each probe.

In silico gene expression profiling - For the *in silico* gene expression profiling the ImmGen consortium [22] data and browsers were used, which were developed as an open resource supported by the National Institute of Allergy and Infectious Diseases (Bethesda, MD, www.immgen.org). ImmGen Gene Skyline tool allows extracting the expression profiles of a selected gene in a group of cell types, basing on fully validated microarray datasets. We here searched for *Lyz2* (lysozyme) or *Emr1* (F4/80) gene expression in "Key populations" data group, or in "Stem and progenitor cells" and "Macrophages (MFs), Neutrophils" for specific subset evaluation. Display settings provide a bar graph with linear scale of expression on x-axis and various cell populations on y-axis.

Western Blotting - Peritoneal macrophages were collected, centrifuged, and lysed on ice in RIPA buffer supplemented with protease inhibitor (Complete PIC, Roche). Protein concentration was estimated for each cell lysate using BCA protein assay (ThermoFisher Scientific). Following protein concentration determination, 40 µg/lane of the prepared protein samples were separated by SDS-polyacrylamide gel electrophoresis. Thereafter, proteins were transferred onto a PVDF membrane (BioRad Laboratories, München, Germany) which was then blocked for 1 hour with 5% non-fat dry milk in Tris-buffered saline containing 0,1% Tween-20 (TBS-T) prior to incubations with antibodies. Primary antibodies against S1P1/EDG-1 (Novus Biologicals, Littleton, CO), phospho and total p70S6-Kinase (Cell Signaling Technologies), phospho and total 4E-BP1 (Cell Signaling Technologies) were used to incubate the blots. Anti-β-actin-peroxidase antibodies (Sigma and Cell Signaling Technologies) were used to visualize actin as a control protein. Antibody binding

visualization was obtained by enhanced chemiluminescence (Western Lightning ECL Pro, Perkin Elmer, Rodgau, Germany), according to the manufacturer's instructions.

Differential leukocyte immunophenotyping – Blood and peritoneal leukocytes were immunophenotyped by flow cytometry as described previously [16,17]. Fluorescently labeled monoclonal antibodies against surface markers on macrophages (F4/80, MHCII, CD86, CD93, CD206, CD115, Dectin-1, MerTK, AXL-1, CD244, CD226) were purchased from ThermoFisher Scientific or BioLegend (Fell, Germany, see Table S2). For each FACS staining 2 x 10^5 cells were incubated with antibody dilutions (0.25 µg for each antibody) in PBS with FBS (1.0 % v/v) for 30 minutes at 4ºC. Cells were fixed for additional 30 minutes with formaldehyde (0.5% v/v). Afterwards, cells were centrifuged for 10 minutes, washed one time in PBS and analyzed on a CyFlow Space flow cytometer (Sysmex Partec, Münster, Germany). For the surface $S1P_1$ detection leukocytes were stained with antibody against EDG1 (1:50, Biorbyt, Cambridge, UK) for 30 minutes at 4°C. After washing, cells were incubated for additional 30 minutes with a secondary antibody (donkey anti-rabbit Alexa Fluor 647, 1:60) and anti-F4/80-PE to identify macrophages. Afterwards, cells were analyzed by flow cytometry. For intracellular staining, peritoneal macrophages were processed using Fix&Perm Cell Permeabilization Kit (ThermoFisher Scientific) following the recommendations of the manufacturer. Briefly, cells were stained with antibodies with F4/80-APC as described above and incubated in the Fixation Medium (Medium A) for 30 min at room temperature. Thereafter, cells were washed with PBS (1x) and the Permeabilization Medium (Medium B, 2x), resuspended in 50 µl of Medium B, which included antibody against IRF8 (PE-conjugated, 0.25 µg/test, ThermoFisher Scientific) and PU1 (Alexa Fluor488-conjugated, 0.25 µg/test, Biolegend). The labelling was carried out at room temperature in the dark for 30 minutes. Subsequently, the cells were washed,

centrifuged, resuspended in PBS supplemented with FBS (1,0% v/v) and analyzed by flow cytometry as described above.

Flow cytometry assessment of kinase activities - Peritoneal leukocytes were isolated as described above and seeded in a 24-well plate at a density of 4,0 x 10^5 cells/mL. After removal of non-adherent cells macrophages were incubated for 0, 60 or 120 minutes in DMEM containing or not S1P (1.0 µmol/L). Subsequently, cells were fixed with pre-warmed BD Phosflow™ Lyse/Fix Buffer I (BD Biosciences, Heidelberg, Germany) for 15 minutes at 37°C. Cells were detached using cell scraper, washed, and permeabilized for 30 minutes at room temperature with BD Phosflow™ Perm/Wash Buffer I (BD Biosciences). Afterwards, cells were stained with anti-F4/80 (APC- or FITC-conjugated, 5.0 µg/mL), anti-phospho-STAT6 (Tyro 641, PE-conjugated, 0.25 µg/mL), anti-phospho-STAT3 (Tyro 705, Alexa Fluor488 -conjugated, 0.25 µg/mL, all from BioLegend) or anti-phospho-AKT (PE-conjugated, 0.25 µg/mL, Miltenyi Biotec, Bergisch Gladbach, Germany) for 30 minutes at room temperature. After washing macrophages were re-suspended in BD Phosflow™ Perm/Wash Buffer I and analyzed using CyFlow Space flow cytometer (Sysmex Partec).

Flow cytometry assessment of apoptosis and efferocytosis – Annexin binding was used as an indicator of macrophage apoptosis. Peritoneal macrophages isolated as described above and seeded in a 24-well plate at a density of 2.5 x 10^5 cells/mL were incubated in DMEM supplemented with FBS (10.0 % v/v) and exposed for 24 h to thapsigargin (0.5 µmol/L) and fucoidan (25 µg/mL) or AcLDL (100 µg/mL) and ACAT inhibitor Sandoz 58-035 (10.0 µg/mL). For determination of the annexin V binding, macrophages were detached from plate by scraping and resuspended in a buffer containing NaCl (140 mmol/L), Hepes (10 mmol/L) and $CaCl_2$ (2.5 mmol/L). Annexin V-FITC (Bender Med-Systems Diagnostics, Vienna, Austria) was added for 30 min at room temperature according to the supplier instructions. Flow cytometric

measurements of annexin V binding were performed on a CyFlow Space flow cytometer (Sysmex Partec). Necrotic cells were detected by counterstaining with propidium iodide (PI). For the assessment of efferocytosis, RAW 264.7 murine macrophages were seeded onto 6 well plate at the density 1 x 10^6 cells/well. Adherent cells were labelled with calcein-AM (1.0 µmol/L, BioLegend) for 30 min and washed twice with PBS to remove dye excess. Apoptotic cells were generated by incubating RAW 264.7 cells with staurosporine (10.0 µmol/L) in serum-free DMEM for 16 - 18 hours. Under these conditions the efficiency of apoptosis exceeded 90 % as tested by Annexin-V staining. Apoptotic cells were collected by scraping, washed in PBS, and resuspended in serum-free DMEM (1 x 10^6 cells/mL). Peritoneal macrophages were isolated as described above, seeded on 12-well plate at density of 0.5 x 10^6 cells/well, and starved overnight in DMEM containing 0.1% (v/v) FBS. Afterwards, cells were washed with PBS and overlayed with apoptotic RAW 264.7 cells at ratio 1:1 (cell/cell). After incubation for 30 min. at 37°C the medium was removed, peritoneal macrophages rinsed twice with ice cold PBS and collected by scraping. The labelling with the APC-conjugated antibody against F4/80 was performed as described above. The fraction of F4/80$^+$ efferocytotic (calcein-positive) cells was assessed by flow cytometry (CyFlow Space).

Fluorimetric assays for caspases 3 and 12 **-** Peritoneal macrophages exposed to thapsigargin or AcLDL as described above were resuspended in a hypotonic cell lysis buffer, subjected to three freeze/thaw cycles, and centrifuged. Caspase activities were measured in the supernatant according to the manufacturer protocols using Ac-DEVD-AFC, LEHD-AFC and ATAD-AFC (380(ex)/500(em) nm), as substrates for caspases 3, 9 and 12, respectively (all from BioVision, Milpitas, CA). Fluorescence was determined using a LS70 spectral fluorimeter (PerkinElmer, Rodgau, Germany). Data

were expressed as relative fluorescence units (RFU) adjusted for the sample protein content.

Cytokine determination – Cytokine (IL-10, IL-4, IL-1RA, IL-5, IL-6, TNF-α, CXCL1/GRO-α) and chemokine (CCL22, CCL5/RANTES) levels were quantified in mouse plasma and supernatants of peritoneal macrophages or neutrophils either by commercially available ELISA (Bio-Techne, see Table S3) or multiplex electrochemiluminescence immunoassay using the V-PLEX Proinflammatory Panel 1 Mouse Kit (MesoScale Discovery, Rockville, ML) according to the manufacturer's instructions.

Determination of the cellular cyclic AMP (cAMP) concentration - Quantification of intracellular cAMP was performed using a DetectX Cyclic AMP Direct EIA Kit (Arbor Assays, Ann Arbor, MI), according to the manufacturer instructions. Briefly, peritoneal macrophages were isolated as described above, seeded in a 48-well plate (4.5 x 10^6 cells/mL) and incubated with 3-isobutyl-1 methyl xanthine (IBMX, Sigma, 0.2 mmol/L) for 30 minutes. After treatment, cells were exposed S1P (1.0 µmol/L) for additional 30 minutes. Cell media were aspirated, and cells were washed with ice-cold PBS. Adherent cells were treated directly with the sample diluent provided by the manufacturer for 10 minutes at room temperature. Cell lysates were precleared by centrifugation (600 x g, 4°C, 15 minutes) and used for EIA as suggested by the manufacturer. The optical density was determined spectrophotometrically at 450nm (FluoSTAR Optima, BMG Labtech, Ortenburg, Germany). Samples were analyzed in duplicate to ensure consistency of reading.

Determination of protein kinase A (PKA) activity – The PKA activity was determined using the PepTag Assay (Promega, Mannheim, Germany) according to the protocol provided by the manufacturer. Briefly, peritoneal macrophages were isolated as described above, seeded on 24-well plate at density of 1 x 10^6 cells/mL and incubated

with or without S1P (1.0 µmol/L) for 2 h. The cells were washed and suspended in PKA extraction buffer containing Tris-HCl (25.0 mmol/L, pH 7.4), EDTA (0.5 mmol/L), EGTA (0.5 mmol/L), β-mercaptoethanol (10.0 mmol/L), leupeptin and aprotinin (each 1.0 µg/mL). After homogenization the lysates were precleared by centrifugation (14000 x g, 5 min, 4°C) and incubated with PKA assay buffer, PKA activator (cAMP, 1.0 µmol/L) and PKA substrate (PepTag® Peptide A, 2.0 µg) for 2h at room temperature. The reaction was stopped by heating the probes (10 minutes, 95°C). Two forms of PKA substrate (phosphorylated and non-phosphorylated) were separated on agarose gel (0,8% (v/v) in 50.0 mmol/L Tris-HCl, pH 8,0) and visualized under UV light. The phosphorylated peptide form was excised from the gel, solubilized using the acidified gel solubilization solution provided by the manufacturer, and its amount was quantified by measuring the absorbance at 570 nm. The negative control containing the PepTag® Peptide A but no kinase was used to determine the exact molar absorptivity of the dye.

Cell cholesterol efflux assay - Peritoneal macrophages were seeded in 48-well plates at a density of 3,5 x 10^5 cells/mL and incubated for 24 h in DMEM containing FBS (10.0 % v/v) and 2 mmol/L glutamine. Cells were labeled with 2.0 µCi/mL [1,2-^{3}H]-cholesterol (Perkin Elmer) in DMEM containing FBS (1.0 % v/v) for 24 hours. Subsequently, cells were treated for 18 hours with 22-(R) hydroxycholesterol (22-OH, 5 µg/mL) and 9-cis retinoic acid (9cRA, 10 µmol/L) or desmosterol (50 µg/mL, Biomol, Hamburg, Germany) in DMEM containing bovine serum albumin (BSA, 2.0 % v/v) and the ACAT inhibitor Sandoz 58-035 (2.0 µg/mL) to prevent cellular accumulation of cholesteryl ester. Cholesterol efflux was induced by adding apoA-I (10.0 µg/mL) or HDL (12.5 µg/mL) for 4 hours. Afterwards, 100 µL of efflux medium was added to scintillation vials containing 4 mL of scintillation cocktail (Ultima Gold, Perkin Elmer) and counted for the radioactivity using beta scintillation spectrometer. Total cholesterol was extracted from macrophages with 0.6 ml of 2-propanol. Lipid extracts were dried under N_2,

resuspended in 1 ml of toluene, and their [^{3}H]-cholesterol content was quantified by liquid scintillation counting. Cholesterol efflux was expressed as a percentage of the radioactivity released into the medium over the total radioactivity incorporated by cells as described previously [23].

Functional characterization of neutrophils - Neutrophils isolated by negative magnetic sorting were counted and seeded in 12-well-plates at a density of 0,4 x 10^5 cells/well. To avoid adherence, Teflon™ sheets were used. Subsequently, cells were stimulated either with lipopolysaccharide (LPS, Sigma, 1.0 µg/mL, 4h), or phorbol-12-myristat-13-acetat (PMA, Sigma, 1.0 µmol/L, 2h). For PMA-stimulated cells dihydrorodamine 123 – a reactive oxygen species (ROS) indicator (DHR, Biomol, 15.0 µmol/L) was added for the last 30 min of incubation. Afterwards, media were collected for cytokine determination and cells were labelled with antibodies against CD62-L (L-selectin shedding indicator), Ly6G, Ly6C and CD11b for flow cytometry.

Statistical procedures - Data are presented as means ± S.D. from at least three independent determinations. The distribution normality was assessed either with Smirnov-Kolmogorov or Shapiro-Wilk tests. Comparisons between two groups were performed with Student t test or Mann-Whitney U test for normally and non-normally distributed populations, respectively. Comparisons between three or more groups were performed with one- or two-way ANOVA with Holm-Sidak test for pairwise *post-hoc* comparisons or Kruskal-Wallis h test with Conover test for pairwise *post-hoc* comparisons for normally and non-normally distributed populations, respectively. p values <0.05 were considered significant.

REFERENCES

1. Clausen BE, et al. Conditional gene targeting in macrophages and granulocytes using LysMcre mice. Transgenic Res 1999;8:265-277
2. Schaller E, et al. Inactivation of the F4/80 glycoprotein in the mouse germ line. Mol Cell Biol. 2002;22:8035-8043.
3. Ishibashi S, et al. Hypercholesterolemia in low density lipoprotein receptor knockout mice and its reversal by adenovirus-mediated gene delivery. J Clin Invest 1993. 92:883-893
4. Effect of apolipoprotein M on high density lipoprotein metabolism and atherosclerosis in low density lipoprotein receptor knock-out mice. J. Biol. Chem. 2008;283,1839-1847
5. Fraenkel-Conrat, H. Methods for investigating the essential groups for enzyme activity. Methods Enzymol. 1957;4:247-269
6. Burkhardt R, et al. Trib1 is a lipid- and myocardial infarction-associated gene that regulates hepatic lipogenesis and VLDL production in mice. J Clin Invest. 2010;120:4410-4414
7. Scherer M, et al. High-throughput analysis of sphingosine 1-phosphate, sphinganine 1-phosphate, and lysophosphatidic acid in plasma samples by liquid chromatography-tandem mass spectrometry. Clin Chem. 2009;55:1218-1222.
8. Daugherty A, Tall AR, Daemen MJAP, Falk E, Fisher EA, García-Cardeña G, Lusis AJ, Owens AP 3rd, Rosenfeld ME, Virmani R; American Heart Association Council on Arteriosclerosis, Thrombosis and Vascular Biology; and Council on Basic Cardiovascular Sciences. Recommendation on design, execution, and reporting of animal atherosclerosis studies: a scientific statement from the American Heart Association. Circ Res. 2017;121:e53-e79

9. Burkhardt R, et al. Cosegregation of aortic root atherosclerosis and intermediate lipid phenotypes on chromosomes 2 and 8 in an intercross of C57BL/6 and BALBc/ByJ low-density lipoprotein receptor-/- mice. Arterioscler Thromb Vasc Biol. 2011;31:775-784
10. Schindelin J, et al. Fiji: an open-source platform for biological-image analysis. Nat Methods 2012;9:676-682
11. Yancey PG, et al. Macrophage LRP-1 controls plaque cellularity by regulating efferocytosis and Akt activation. Arterioscler Thromb Vasc Biol. 2010;30:787-795.
12. Schrijvers DM, et al. Phagocytosis of apoptotic cells by macrophages is impaired in atherosclerosis. Arterioscler Thromb Vasc Biol. 2005;25:1256–1261.
13. Thorp E, et al. Mertk receptor mutation reduces efferocytosis efficiency and promotes apoptotic cell accumulation and plaque necrosis in atherosclerotic lesions of apoe-/- mice. Arterioscler Thromb Vasc Biol. 2008;28:1421–1428.
14. Yam LT et al. Cytochemical identification of monocytes and granulocytes. Am J Clin Pathol 1971;55:283-290
15. Vorbach S, et al. F. Enhanced expression of the sphingosine-1-phosphate-receptor-3 causes acute myelogenous leukemia in mice. Leukemia. 2020 Mar;34(3):721-734
16. Potì F, et al. KRP-203, sphingosine 1-phosphate receptor type 1 agonist, ameliorates atherosclerosis in LDL-R−/− mice. Arterioscler Thromb Vasc Biol. 2013;33:1505–1512.
17. Nofer JR, et al. FTY720, a synthetic sphingosine 1 phosphate analogue, inhibits development of atherosclerosis in low-density lipoprotein receptor–deficient mice. Circulation. 2007;115:501–508
18. Kallio MA, et al. Chipster: user-friendly analysis software for microarray and other high-throughput data. BMC Genomics. 2011;12:507

19. Smyth GK. Linear models and empirical bayes methods for assessing differential expression in microarray experiments. Stat Appl Genet Mol Biol. 2004;3:Article 3.
20. Zhou Y, et al. Metascape provides a biologist-oriented resource for the analysis of systems-level datasets. Nat Commun. 2019;10:1523.
21. Blecher-Gonen R, et al. High-throughput chromatin immunoprecipitation for genome-wide mapping of in vivo protein-DNA interactions and epigenomic states. Nat Protoc. 2013;8:539-554
22. Heng TS, Painter MW; Immunological Genome Project Consortium. The Immunological Genome Project: networks of gene expression in immune cells. Nat Immunol. 2008;9:1091-1094.
23. Zanotti I, et al. The LXR agonist T0901317 promotes the reverse cholesterol transport from macrophages by increasing plasma efflux potential. J Lipid Res. 2008;49:954-960
24. Rébé C, et al. Identification of biological markers of liver X receptor (LXR) activation at the cell surface of human monocytes. PLoS One. 2012;7(11):e48738

SUPPLEMENTAL FIGURES

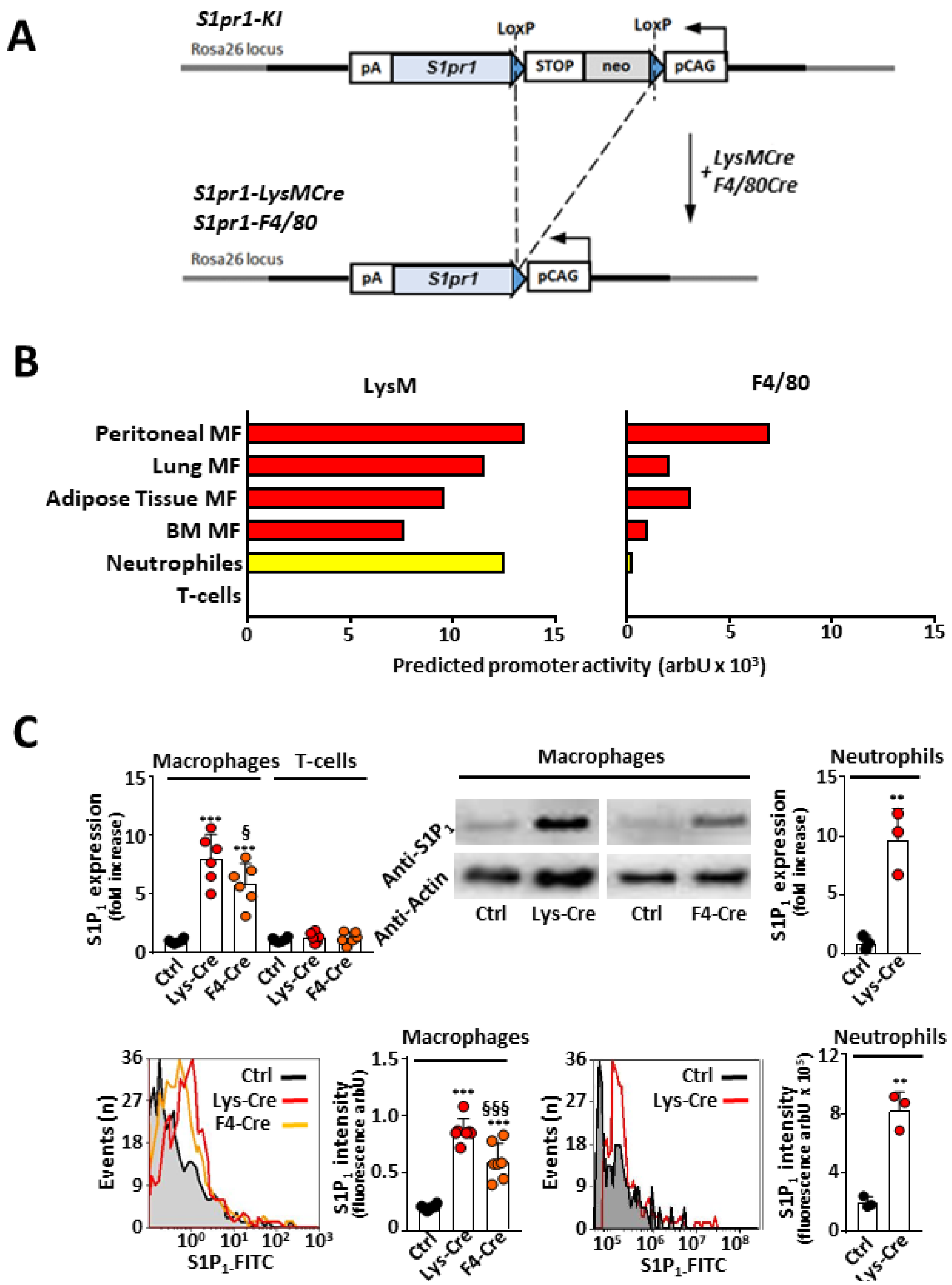

***Supplemental Figure 1**. Generation of S1pr1-LysMCre or S1pr1-F4/80Cre mice and characterization of $S1P_1$ overexpressing macrophages and neutrophils* - **A**. Schematic representation of the targeting vector prior to (top) and following (bottom) Cre recombination. The excision of a floxed blocking element activates pCAG promoter and $S1P_1$ expression **B**. Predicted LysM and F4/80 promoter activities in myeloid cells as determined with ImmGen Skyline tool. **C**. Peritoneal MF or splenocytes were collected from *S1pr1-KI* (Ctrl), *S1pr1-LysMCre* (Lys-Cre) or *S1pr1-F4/80Cre* (F4-Cre) fed Chow diet. Neutrophils were isolated from bone marrows by magnetic sorting. *S1p1* expression was analyzed by qPCR. mRNA levels were normalized to *Gapdh* and presented relative to *S1p1-KI*. Protein extracts were subjected to Western Blotting with an anti-$S1P_1$ or anti-beta-actin antibody. Cell surface staining for $S1P_1$ was analyzed by flow cytometry. Shown are representative histograms superimposed for comparison and bar graphs representing fluorescent medians. Data represent means ± SD from 3 to 6 determinations. Blots are representative for one experiment out of two. * - $p<0.05$, ** - $p<0.01$, *** - $p<0.001$ (Lys-Cre vs. Ctrl or F4-Cre vs. Ctrl, ANOVA except **C** S1P intensity/Neutrophils: Kruskal-Wallis h-test). MF – macrophages.

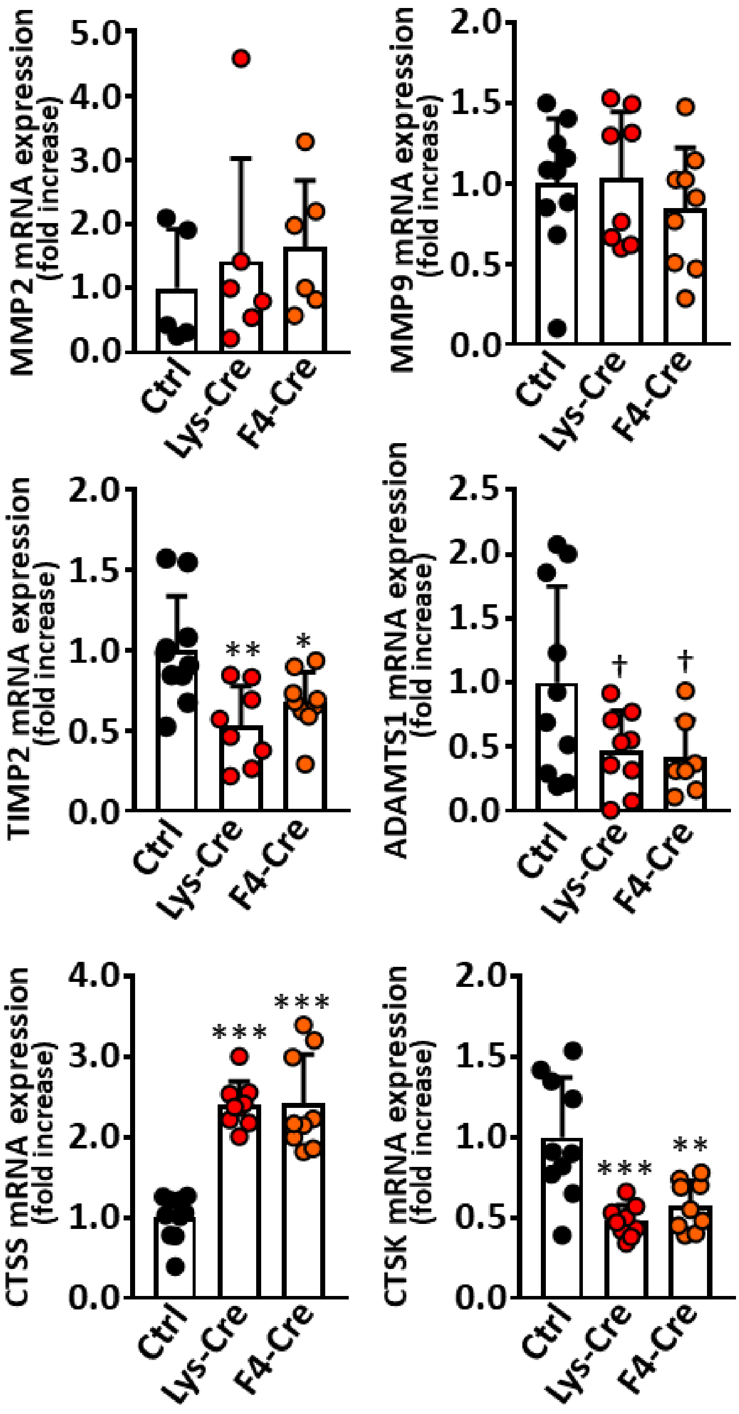

Supplemental Figure 2. *Effect of S1P$_1$ overexpression in macrophages on the expression of collagen-degrading proteinases* – Peritoneal macrophages were collected from *Ldlr*$^{-/-}$ mice transplanted with *S1pr1-KI* (n=6-10), *S1pr1-LysMCre* (n=6-9) or *S1pr1-F4/80Cre* (n=6-9) BM and fed Western diet (WD). The expressions of matrix metalloproteinases (*Mmp*) 2 and 9, a disintegrin and metalloproteinase with a thrombospondin type 1 motif (*Adamts*) member 1, tissue inhibitor of metalloproteinase (*Timp*) 2 and cathepsins (*Cts*) S and K were analyzed by qPCR. mRNA levels were normalized to *Gapdh* and presented relative to *S1pr1-KI*. † - p<0.1, * - p<0.05, ** - p<0.01, *** - p<0.001 (Lys-Cre vs. Ctrl or F4-Cre vs. Ctrl, ANOVA except *Mmp2*mRNA expression: Kruskal-Wallis h-test).

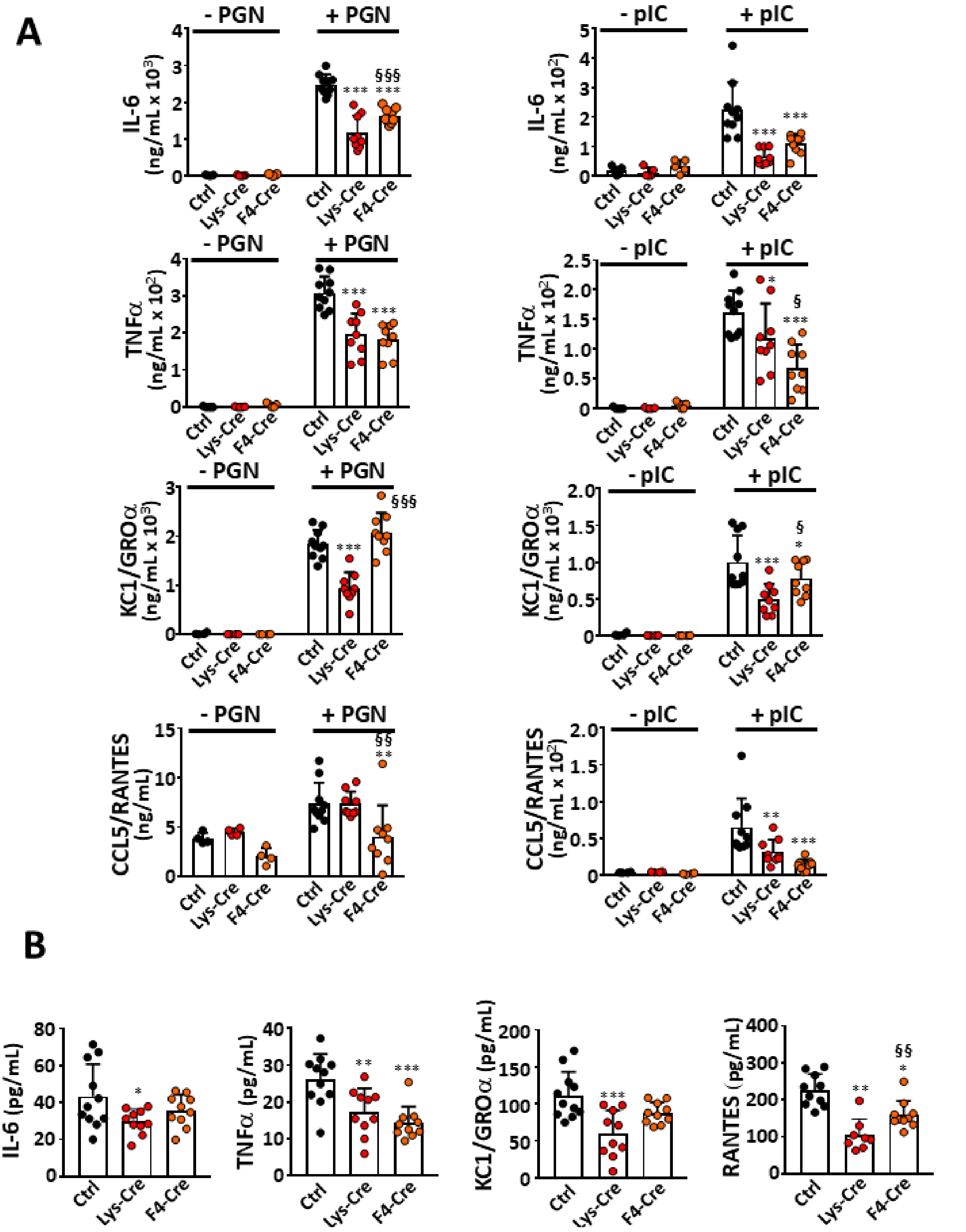

***Supplemental Figure 3**. Effect of S1P$_1$ overexpression in macrophages on pro-inflammatory cyto- and chemokine production* – **A.** Peritoneal macrophages from either *S1pr1-KI* (Ctrl, n=4-10), *S1pr1-LysMCre* (Lys-Cre, n=4-9) or *S1pr1-F4/80Cre* (F4-Cre, n=4-9) mice on normal diet were incubated for 24 h in media containing agonists of TLR2 (peptidoglycan, PGN, 0.02 µg/mL) or TLR3 (polyinosinic–polycytidylic acid, pIC, 0.05 µg/mL). Cyto- and chemokines in medias were determined by ELISA. **B**. Cyto- or chemokines in plasmas from LDL-R$^{-/-}$ mice on WD transplanted with *S1pr1-KI* (n=10-11), *S1pr1-LysMCre* (n=8-10) or *S1pr1-F4/80Cre* (n=10) BM were determined by V-PLEX Proinflammatory Panel 1 Mouse Kit (MesoScale).* - p<0.05, ** - p<0.01, *** - p<0.001 (Lys-Cre vs. Ctrl or F4-Cre vs. Ctrl) § - p<0.05, §§ - p<0.01, §§§ - p<0.001 (Lys-Cre vs. F4-Cre, ANOVA except **B** RANTES: Kruskal-Wallis h-test).

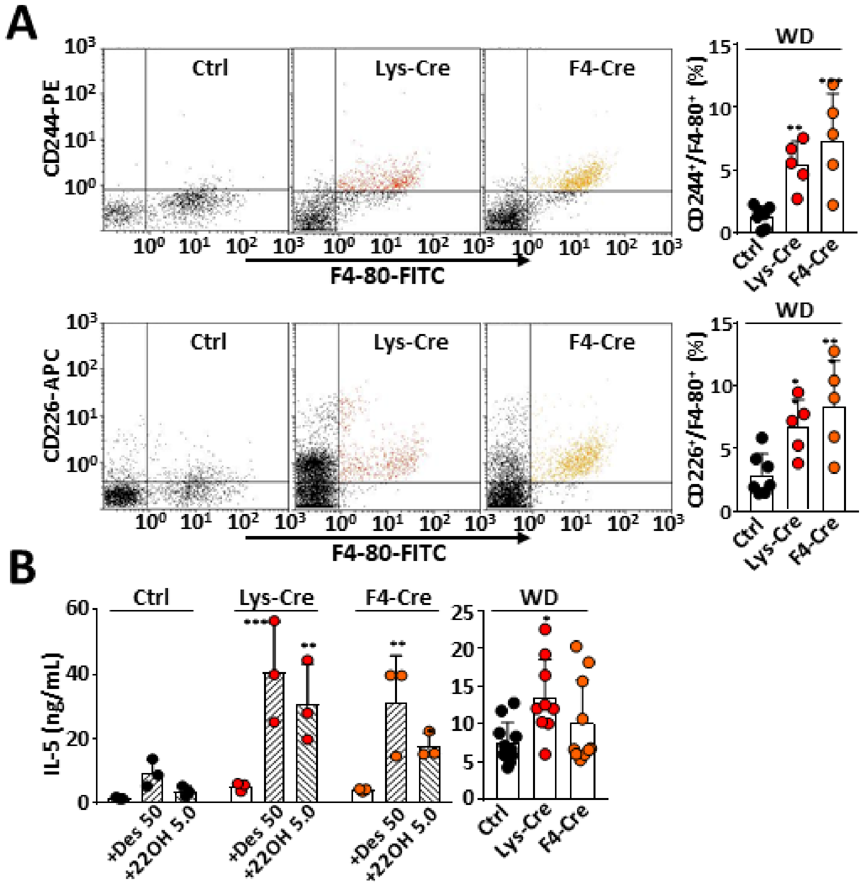

***Supplemental Figure 4**. Additional phenotypic characterization of S1P$_1$ overexpressing macrophages* - Peritoneal macrophages were collected from LDL-R$^{-/-}$ mice transplanted with *S1pr1-KI* (n=11), *S1pr1-LysMCre* (n=9) or *S1pr1-F4/80Cre* (n=10) BM and fed Western diet (WD). **A**. Cell surface staining for LXR activity markers CD244 and CD226 [24] was analyzed by flow cytometry. Shown are representative dot-blots and bar graphs representing percent of CD244$^+$/F4/80$^+$ and CD226$^+$/F4/80$^+$ macrophages (n=5-7 pro group). **B**. Peritoneal macrophages (n=3 pro group) established in cell culture were incubated for 24 h in media containing desmosterol (Des, 50 µmol/L) or 22-hydroxycholesterol and 9-cis-retinoic acid (22OH, 5.0 µmol/L). IL-5 concentrations in cell media and plasmas from WD-fed mice were determined by ELISA. * - $p<0.05$, ** - $p<0.01$, *** - $p<0.001$ (Lys-Cre vs. Ctrl or F4-Cre vs. Ctrl, ANOVA except **B** IL-5/WD Kruskal-Wallis h-test).

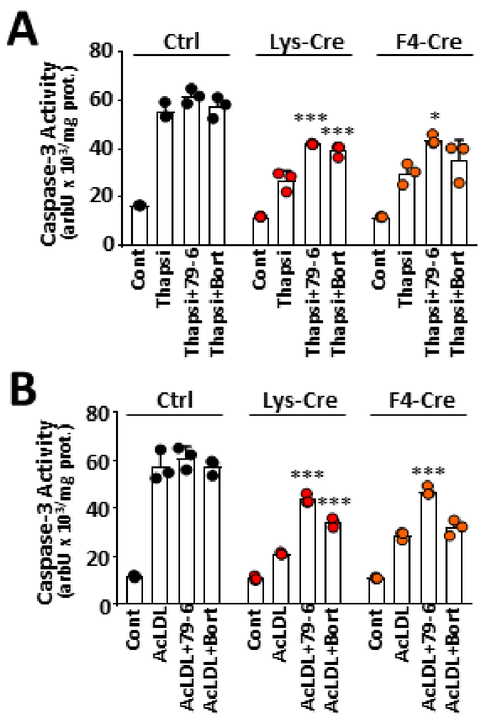

***Supplemental Figure 5**. Effect of modulators of anti-apoptotic proteins on thapsigargin- or cholesterol-loading-induced caspase 3 activity* - Peritoneal macrophages from either *S1pr1-KI* (Ctrl, n=3), *S1pr1-LysMCre* (Lys-Cre, n=3)) or *S1pr1-F4/80Cre* (nF4-Cre, n=3) mice on normal diet were established in cell culture and incubated for 30 min with 79-6 (Bcl6 inhibitor, 50.0 µmol/L), bortezomib (Maf inhibitor, 100 nmol/L) or vehiculum (Cont) prior to adding thapsigargin/fukoidan (Thapsi, 0.5 µmol/L and fukoidan 25.0 µg/mL, **A**) or acetylated LDL (AcLDL, 100.0 µg/mL, **B**) for 24 h. Bar graphs show caspase-3 activities (lower panels). * - p<0.05, ** - p<0.01 (thapsigargin vs. thapsigargin + inhibitor or AcLDL vs. AcLDL + inhibitor, ANOVA).

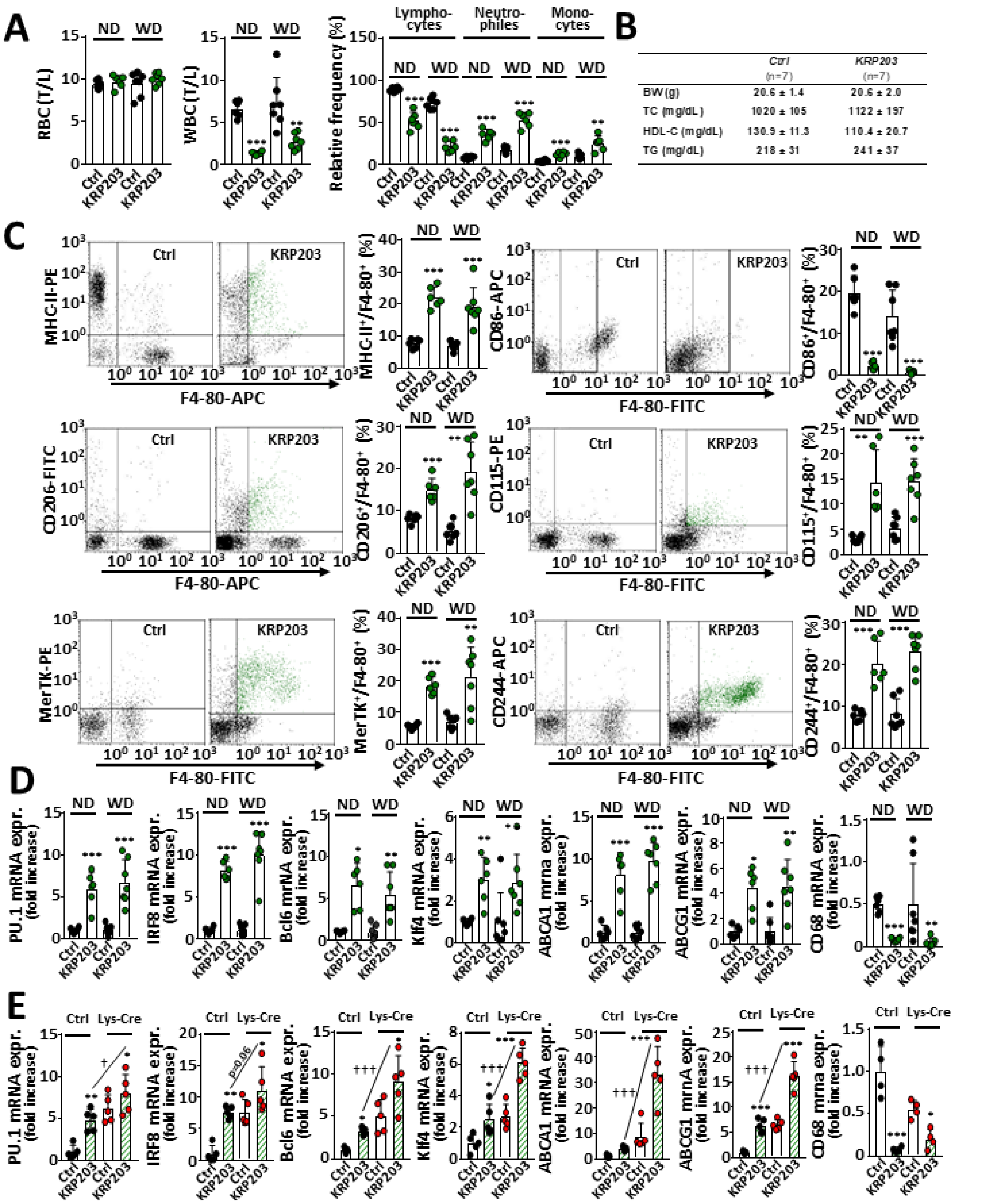

	Ctrl (n=7)	*KRP203* (n=7)
BW (g)	20.6 ± 1.4	20.6 ± 2.0
TC (mg/dL)	1020 ± 105	1122 ± 197
HDL-C (mg/dL)	130.9 ± 11.3	110.4 ± 20.7
TG (mg/dL)	218 ± 31	241 ± 37

Supplemental Figure 6. *Effect of $S1P_1$ agonist KRP-203 on peripheral blood counts and macrophage phenotype in WT or LDL-$R^{-/-}$ mice* - ND-fed *WT* or *Ldlr*$^{-/-}$ mice fed WD for 4 weeks were administered KRP-203 (3.0 mg/kg, ND: n=6, WD: n=6) or saline (controls (Ctrl), ND: n=7, WD: n=7) for further 4 weeks, euthanized, and blood and PM were collected. **A.** Red and white blood cell counts (RBC, WBC) and differential leukocyte counts were determined on hematology analyzer. **B**. Body weights (BW), plasma total cholesterol (TC), HDL cholesterol (HDL-C) and triglycerides (TG) were determined in *Ldlr*$^{-/-}$ mice. **C** and **D**. PM were directly analyzed by flow cytometry or established in cell culture. (C) Cell surface stainings for MHC-II, CD115, CD206, CD86, MerTK and CD244. Bar graphs represent percent of cells positive for each marker and F4/80. (D) *Sp1* (PU.1), *Irf8*, *Bcl6*, *Klf4*, *Abca1*, *Abcg1* and *Cd68* expression analyzed by qPCR. mRNA levels were normalized to *Gapdh* **E**. PM obtained from *S1p1-KI* mice (Ctrl, n=5 pro group)) *or S1pr1-LysMCre* mice overexpressing $S1P_1$ (Lys-Cre, n=5 pro group) were exposed for 24 h to KRP-203-phosphate (1.0 µmol/L). Gene expression was analyzed and presented as described above. * - p<0.05, ** - p<0.01, *** - p<0.001 (KRP-203 vs. Ctrl), † - p<0.05, ††† - p<0.001 (Ctrl vs. Lys-Cre, Student t test except **C** CD115/ND, **C** CD244/WD, **D** *Klf4*/WD, **D** *Abcg1*/WD, **D** Cd68/WD: Mann-Whitney U test).

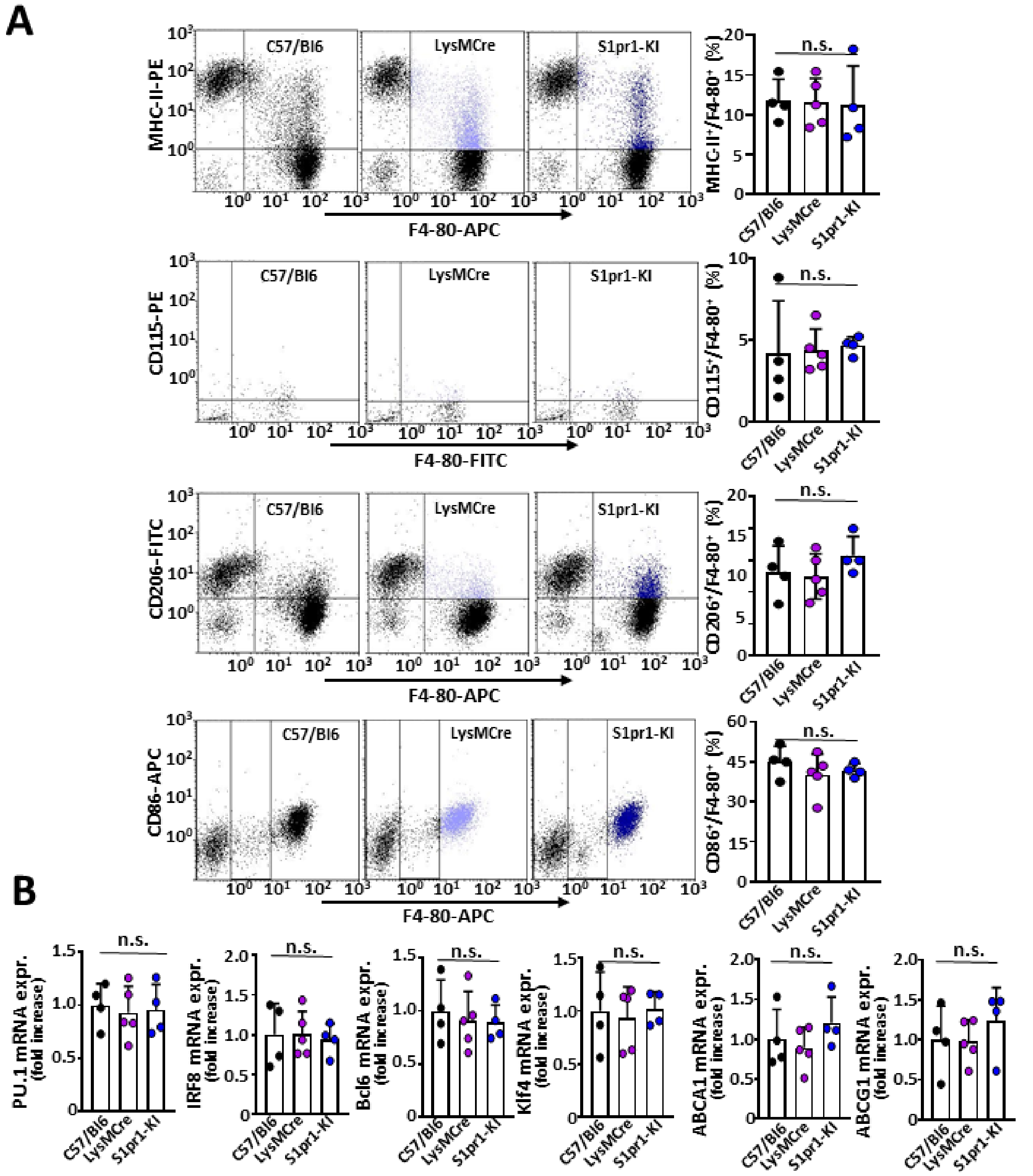

Supplemental Figure 7. *Effect of mouse line on PM phenotype* - ND-fed WT (*C57/Bl6*) mice (n=5), *S1pr1-KI* mice (n=4) and *LysMCre* mice (n=5) were euthanized, and peritoneal macrophages were collected, directly analyzed by flow cytometry, or established in cell culture. **A.** Cell surface stainings for MHC-II, CD115, CD206 and CD86. Bar graphs represent percent of cells positive for each marker and F4/80. **B**. *Sp1* (PU.1), *Irf8*, *Bcl6*, *Klf4*, *Abca1* and *Abcg1* expression analyzed by qPCR. mRNA levels were normalized to *Gapdh* and are presented relative to controls. n.s. – not significant (Kruskal-Wallis h-test).

A

	S1pr1-KI/apoM$^{-/-}$ (n=9)	*S1pr1-LysMCre/apoM*$^{-/-}$ (n=8)
BW (g)	22.3 ± 2.0	21.5 ± 1.7
TC (mg/dL)	826 ± 317	462 ± 124*
HDL-C (mg/dL)	86.0 ± 12.8	62.0 ± 12.5*
TG (mg/dL)	275 ± 114	130 ± 35.4*
S1P (µmol/L)	0.89 ± 0.19	1.24 ± 0.36

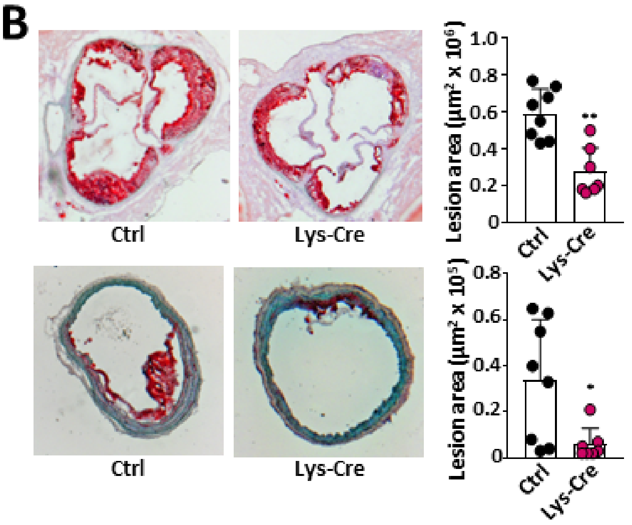

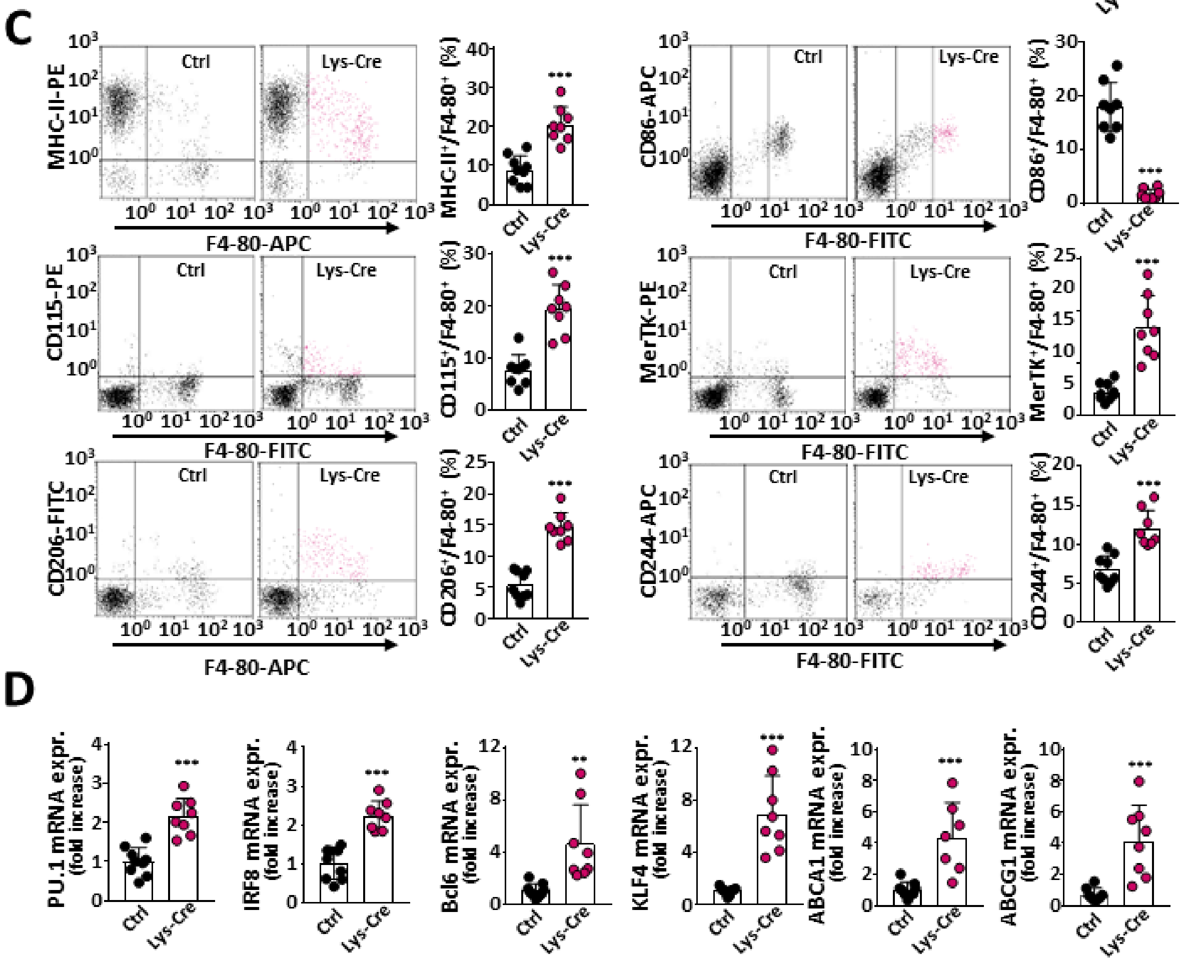

Supplemental Figure 8. – Effect of apoM deficiency on plasma lipids, atherosclerosis and macrophage phenotype in LDL-R$^{-/-}$ mice with hematopoietic S1P$_1$-overexpression – *ApoM*$^{-/-}$/*LDL-R*$^{-/-}$ mice were transplanted with bone marrow from *S1pr1-KI* (Ctrl, n=8-9) or *S1pr1-LysMCre* (Lys-Cre, n=7-8) mice and fed Western diet (WD) for 14 weeks. Animals were euthanized and aortas, brachiocephalic arteries, blood, and peritoneal macrophages were collected, **A**. Plasma lipids and S1P concentrations were determined as described under Methods **B**. Aortic roots and brachiocephalic arteries were used for morphometric analysis. Shown are representative Oil Red O stainings of lesions and quantification of lesion areas (bar graphs). **C**. and **D**. Peritoneal macrophages were directly analyzed by flow cytometry or established in cell culture. (C) Cell surface stainings for MHC-II, CD115, CD206, CD86, MerTK and CD244. Bar graphs represent percent of cells positive for each marker and F4/80. (D) *Sp1* (PU.1), *Irf8*, *Bcl6*, *Klf4*, *Abca1* and *Abcg1* expression analyzed by qPCR. mRNA levels were normalized to *Gapdh* and are presented relative to controls. * - p<0.05, ** - p<0.01, *** - p<0.001 (Lys-Cre vs. Ctrl, Student t test except **B** upper panel, **D** *Bcl6* mRNA: Mann-Whitney U test).

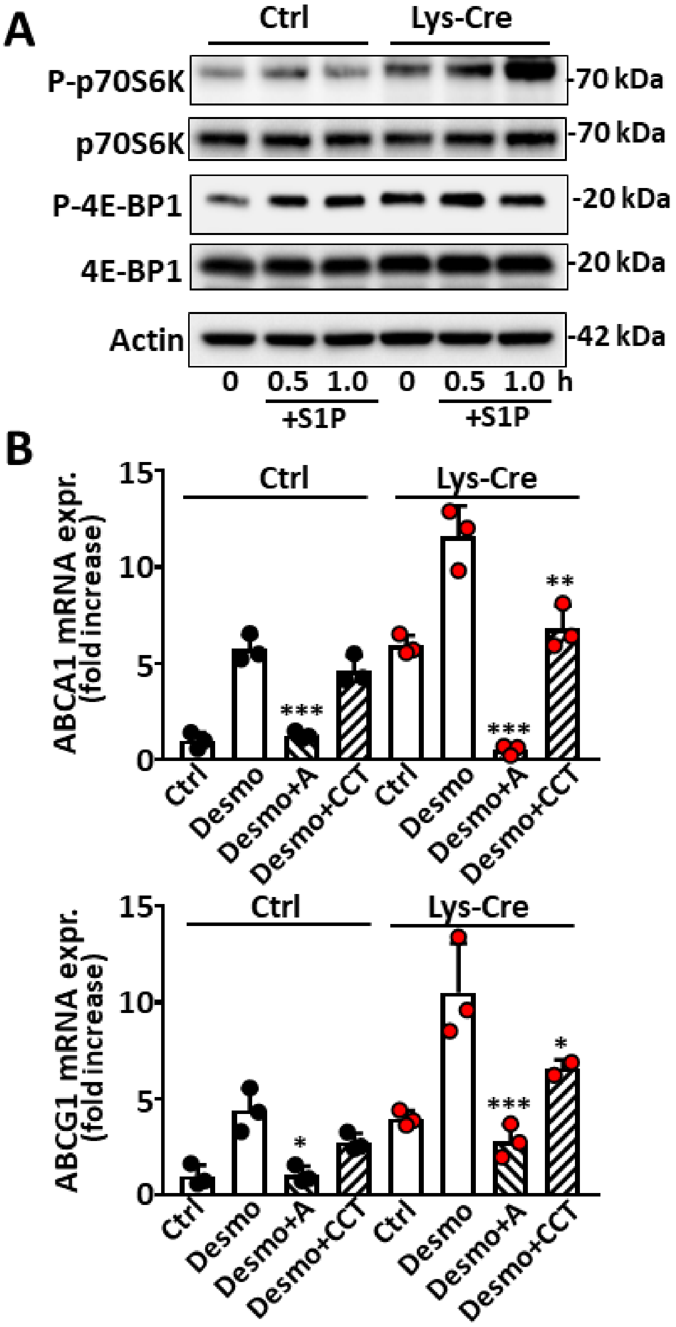

Supplemental Figure 9*. Stimulatory effects of S1P on mTOR pathway and the involvement of Akt isoforms* – PM from *S1pr1-KI* (Ctrl) or *S1pr1-LysMCre* (Lys-Cre) mice fed ND were established in culture. **A**. PM were exposed to S1P (0.5 and 1.0 µmol/L) for indicated times. Cell lysates were probed with antibodies against total and phosphorylated (P) p70S6 kinase or 4E-BP1. Blots are representative for 2 independent experiments. **B**. Cells (n=3 pro group) were exposed for 30 min to inhibitors of Akt1 A-674563 (A, 50.0 nmol/L) or Akt2 CCT-128930 (CCT, 10.0 µmol/L) prior to incubation with desmosterol (50 µmol/L) for 24 h. *Abca1* and *Abcg1* genes analyzed by qPCR. * - p<0.05, ** - p<0.01, *** - p<0.001 with vs. without treatment with inhibitor (ANOVA).

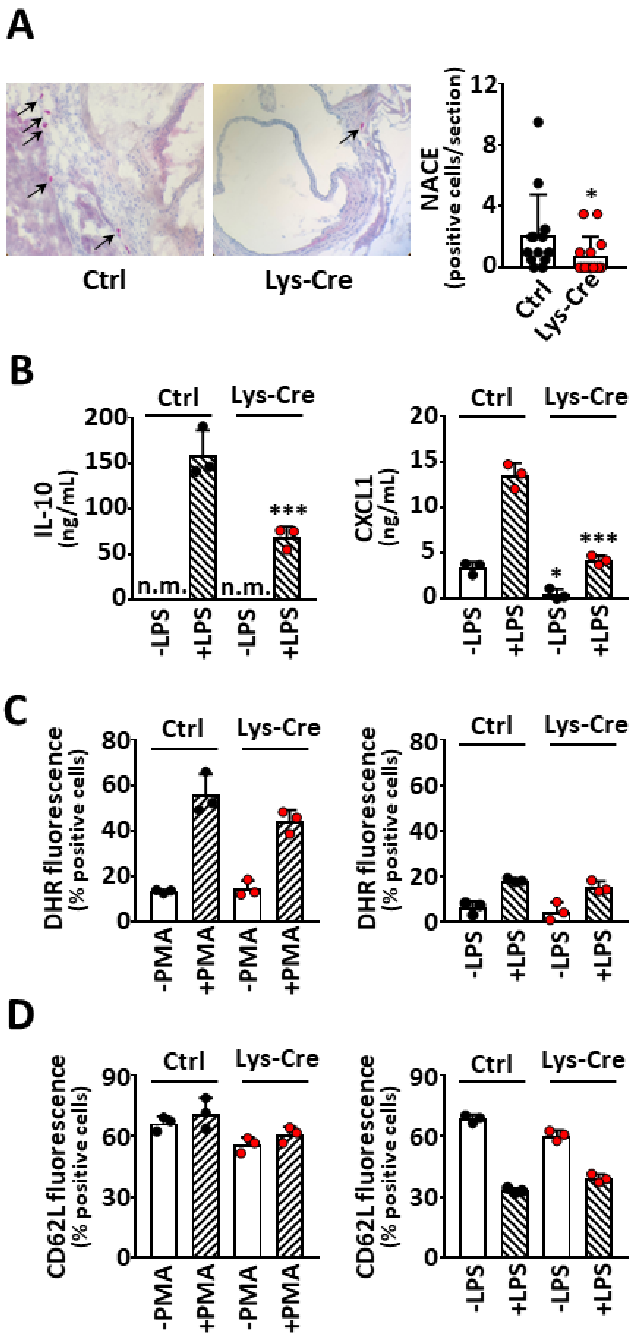

***Supplemental Figure 10**. Effect of $S1P_1$ overexpression in neutrophils on their atherosclerotic plaque content and activation* – **A.** Aortic root sections from Western diet-fed LDL-R$^{-/-}$ mice transplanted with *S1pr1-KI* (Ctrl, n=11) or *S1pr1-LysMCre* (Lys-Cre, n=10) bone marrow was stained for neutrophils as described under Methods. Bar graphs show the neutrophil content in plaques expressed as the percentage of lesion area. **B - D**. Neutrophils isolated from *S1pr1-LysMCre* (Lys-Cre, n=4 animals) or *S1pr1-KI* (Ctrl, n=3 animals) bone marrows were pooled and exposed to lipopolysaccharide (LPS, 1.0 µg/mL, 3 h) or phorbol-12-myristat-13-acetat (PMA, 1.0 µmol/L, 12 h). Cyto- or chemokine production (**B**), reactive oxygen species (ROS) production (**C**) and L-selectin (CD62L) shedding (**D**) were determined as described in Methods. Experiments were performed in triplicates. * - p<0.05, ** - p<0.01 *** - p<0.001 (Lys-Cre vs. Ctrl, one-way ANOVA except **A** Mann-Whitney U test).

SUPPLEMENTAL TABLES

Supplemental Table 1. Red and white blood cell parameters and leukocyte distribution in mice overexpressing $S1P_1$

Parameter	***S1pr1-KI***		***S1pr1-LysMCre***		***S1pr1-F4/80Cre***	
	**ND (n=8-10)**	**WD (n=11)**	**ND (n=9-10)**	**WD (n=10)**	**ND (n=9-10)**	**WD (n=10)**
Red blood cells (T/L)	9.5 ± 0.9	8.2 ± 3.4	5.4 ± 0.6***	3.6 ± 0.8***	8.0 ± 1.4**	5.5 ± 0.9***
Hemoglobin (g/dL)	14.6 ± 1.4	12.3 ± 1.1	10.0 ± 1.4***	5.3 ± 1.7***	13.3 ± 1.0	9.2 ± 1.6***
Hematocrit (%)	48.5 ± 5.8	43.1 ± 4.2	33.3 ± 4.2***	22.5 ± 5.7***	44.1 ± 6.7	34.3 ± 5.6***
Mean Corpuscular Volume (fL)	50.3 ± 4.0	52.9 ± 1.3	61.4 ± 4.3***	77.5 ± 7.6***	55.5 ± 1.6**	61.4 ± 2.6***
Mean Corpuscular Haemoglobin (pg)	15.2 ± 1.5	15.2 ± 0,4	18.4 ± 1.8`**	17.1 ± 1.0***	17.0 ± 2.1	16.7 ± 0.4***
Mean Corpuscular Hemoglobin Concentration (g/dL)	30.3 ± 1.9	28.7 ± 0.9	30.1 ± 3.7	22.3 ± 2.8***	30.6 ± 3.0	27.2 ± 0.8***
White blood cells (G/L)	3.6 ± 1.0	8.2 ± 3.3	4.9 ± 2.0	10.4 ± 2.7	4.0 ± 1.3	9.0 ± 1.9
Neutrophils (%)	8.1 ± 2.5	7.8 ± 2.7	19.2 ± 11.0*	10.7 ± 4.3	10.8 ± 4.1	9.2 ± 3.2
Monocytes (%)	1.9 ± 1.8	4.5 ± 2.1	8.4 ± 2.3***	15.6 ± 5.6***	2.0 ± 1.4	5.2 ± 1.2
Lymphocytes (%)	89.1 ± 4.4	87.0 ± 2.5	71.7 ± 12.9***	73.6 ± 5.2***	86.8 ± 5.5	85.6 ± 3.5
Platelets (G/L)	1251 ± 381	611 ± 246	1002 ± 587	230 ± 154**	1162 ± 226	570 ± 291
$Ly6G^+/CD11b^+$ (%)	5.0 ± 2.6	4.5 ± 2.1	14.7 ± 6.2***	13.3 ± 3.7***	4.9 ± 2.0	4.4 ± 1.6
$CD115^+/CD11b^+$ (%)	5.1 ± 1.8	5.4 ± 1.9	14.0 ± 5.1***	13.7 ± 5.3***	5.7 ± 1.1	5.5 ± 1.5
$B220^+$ (%)	35.1 ± 11.2	57.2 ± 4.1	31.3 ± 8.1	51.0 ± 5.8	31.2 ± 6.5	56.1 ± 4.4
$CD3^+$ (%)	37.2 ± 6.1	26.4 ± 3.9	28.6 ± 3.6*	23.2 ± 5.5	40.2 ± 8.6	24.4 ± 4.3
$CD4^+/CD3^+$ (%)	64.8 ± 4.5	46.9 ± 3.7	63.6 ± 2.5	46.9 ± 3.5	61.3 ± 3.3	49.3 ± 5.7
$CD8^+/CD3^+$ (%)	32.0 ± 4.2	49.9 ± 4.2	33.5 ± 2.1	48.0 ± 4.3	34.0 ± 3.2	47.4 ± 5.4

Data represent means ± SD. * - p<0.05, ** - p<0.01, *** - p<0.001 (S1pr1-LysMCre vs. S1pr1-KI or S1pr1-F4/80Cre vs. S1pr1-KI, ANOVA except for RBC/WD, WBC/ND, MCV/WD, MCHC/ND, MCHC/WD and Monocytes/ND: Kruskal-Wallis h-test). ND – normal diet. WD – Western diet. For neutrophils, lymphocytes, and monocytes from S1pr1-LysMCre mice on WD n=6.

Supplemental Table 2. Body weights and plasma lipid concentrations

	S1pr1-KI (n=11)	***S1pr1-LysMCre*** (n=10)	***S1pr1-F4/80Cre*** (n=10)
Body weight (g)	22.9 ± 2.0	21.2 ± 1.6	22.3 ± 1.4
Total cholesterol (mg/dL)	1373 ± 247	1351 ± 355	1309 ± 320
HDL-Cholesterol (mg/dL)	87.1 ± 13.6	94.6 ± 12.1	84.5 ± 17.8
Triglycerides (mg/dL)	360 ± 128	330 ± 111	292 ± 106
S1P (µmol/L)	2.19 ± 0.47	1.83 ± 0.60	1.63 ± 0.65

Data represent means ± SD. For S1P n=6

Supplemental Table 3. List of PCR primers used in the study

Gene	Forward primer	Reverse primer
	Expression studies	
Abca1	GGACATGCACAAGGTCCTGA	CAGAAAATCCTGGAGCTTCAAA
Abcg1	CTGAAAAGAATGGGTGTTGG	ACCTGGACAGGAAAGAATCC
Adamts1	CATAACAATGCTGCTATGTGCG	TGTCCGGCTGCAACTTCAG
Arg1	CGATTCACCTGAGCTTTGAT	AAGCCAAGGTTAAAGCCACT
Axl1	GAAGGTCAGCTCAATCAGGA	GTCAGAGCCCTGAAAACAGA
Bcl6	CACACCCGTCCATCATTGAA	TGTCCTCACGGTGCCTTTTT
Cebpa (C/EBPα)	GCGGGAACGCAACAACATC	GTCACTGGTCAACTCCAGCAC
Cd163	GCAAAAACTGGCAGTGGG	GTCAAAATCACAGACGGAGC
Cd68	TGTCTGATCTTGCTAGGACCG	GAGAGTAACGGCCTTTTTGTGA
Chil3 (YM1)	CCAGCAGAAGCTCTCCAGAAGCA	TGGTAGGAAGATCCCAGCTGTACG
Ciita (MHC-II)	GACGCTCAACTTGTCCCAAAAC	GCAGCCGTGAACTTGTTGAAC
Csf1r (CD115)	TGGATGCCTGTGAATGGCTCTG	GTGGGTGTCATTCCAAACCTGC
Ctsc	GCCATTCCTCCTTCTTCTTC	CTAGCAATTCCGCAGTGATT
Ctsk	CATGGTGAGCTTTGCTCTGT	CCAGGTTATGGGCAGAGATT
Egr1	ACAGCAGTCCCATCTACTCG	CTCCCTGTTGTTGTGGAAAC
Retnla (Fizz-1)	CCAATCCAGCTAACTATCCCTCC	ACCCAGTAGCAGTCATCCCA
Gapdh	CTGGAGAAACCTGCCAAGTA	TGTTGCTGTAGCCGTATTCA
Gas6	CCTACCAAGTCTTCGAGGAG	CACATTTGGCGAAATCTGGG
Hmox1	TGATGGCTTCCTTGTACCAT	CTCGTGGAGACGCTTTACAT
Il5	CTGGCCTCAAACTGGTAATG	TGAGGGGGAGGGAGTATAAC
Il10	TGTCAAATTCATTCATGGCCT	ATCGATTTCTCCCCTGTGAA
Irf8	AGACCATGTTCCGTATCCCCT	CACAGCGTAACCTCGTCTTCC
Klf4	TCAAGTTCCCAGCAAGTCAG	AAACTTCCAGTCACCCCTTG
Lgmn	TGCTACCAGGAGGCTGTAAC	TTGTCCATGGCCATCTCTAT
Nr1h3 (LXRα)	TGAGAGCATCACCTTCCTCA	TGGAGAACTCAAAGATGGGG
Nr1h2 (LXRβ)	ATTAAGGAAGAGGGGCAGGA	GCTGAGCACGTTGTAGTGGA
Mafb	GAGCGAGCAGAGTTTCAGTC	AGCTTGCTGCTACCTTCTCA
Mertk	CGCCAAGGCCGCCTT	TCGGTCCGCCAGGCT
Mmp2	CAAGTTCCCCGGCGATGTC	TTCTGGTCAAGGTCACCTGTC
Mmp9	CTGGACAGCCAGACACTAAAG	CTCGCGGCAAGTCTTCAGAG
Selplg (PSGL-1)	CTTCCTTGTGCTGCTGACCAT	TCAGGGTCCTCAAAATCGTCATC
Spi1 (PU.1)	CCTACATGCCCCGGATGTGC	TGCTGTCCTTCATGTCGCCG
S1pr1	TTCCATCTGCTGCTTCATCATCC	GGTCCGAGAGGGCTAGGTTG
Timp2	TCAGAGCCAAAGCAGTGAGC	GCCGTGTAGATAAACTCGATGTC
	Chromatin immunoprecipitation studies	
**Abca1 promoter**	GGGGAAAGAGGGAGAGAACAG	GAATTACTGGTTTTTGCCGC
**Abcg1 promoter**	CCATTAGCTGACTGTGAGCAT	GGGCAGGCAAGTGGTTGTCACAT
**Arg1 promoter**	TGCTCCGTTTCGATTCTTCT	TCGTGTGCCAAGTGCTATTC
**Ciita promoter**	CCTAACCATTTCCGTTCATC	ACCTCTGTTGTCAGTGTAGCCTTCT
**Csf1r promoter**	CTGCTGCTGGCCACAGTTT	CAGCGATGCCCTCTTTGC
**Il5 promoter**	TCCGCCATATATGCACAACTT	TTAACATCTTGACCCCCACCC

Supplemental Table 4. List of fluorescent-labeled antibodies used in the study

Antibody	Chromophore	Ordering Number	Supplier
**PU.1**	AlexaFluor488	681305	BioLegend
**IRF8**	PE	12-9852-82	eBioscience (ThermoFisher)
**MHCII**	PE	107607	BioLegend
**CD115**	PE	135505	BioLegend
**F4/80**	FITC	123107	BioLegend
	APC	123115	BioLegend
**CD206**	FITC	141703	BioLegend
**CD86**	APC	105011	BioLegend
**CD93**	APC	136509	BioLegend
**PU.1**	AlexaFluor488	681305	BioLegend
**IRF8**	PE	12-9852-82	eBioscience (ThermoFisher)
**MerTK**	PE	151505	BioLegend
**Axl1**	AlexaFluor647	51-1621-82	eBioscience (ThermoFisher)
**pAKT**	PE	12-9715-42	eBioscience (ThermoFisher)
**pSTAT3**	AlexaFluor488	651005	BioLegend
**pSTAT6**	PE	12-9013-42	eBioscience (ThermoFisher)
**CD244**	FITC	133503	BioLegend
**CD226**	PE	128805	BioLegend
**CD36**	APC	102611	BioLegend
**CD4**	FITC	100509	BioLegend
**CD3**	PE	100205	BioLegend
**CD8a**	APC	100711	BioLegend
**CD11b**	PE	101207	BioLegend
	APC-Cy7	101225	BioLegend
	BV 421	101235	BioLegend
	FITC	101205	BioLegend
**B220**	APC-Cy7	103223	BioLegend
**Ly6G**	APC-Cy7	127623	BioLegend
	PE	127608	BioLegend
	FITC	127606	BioLegend
**Ly6C**	AlexaFluor647	128009	BioLegend
	PE	128007	BioLegend
**CD62**	APC	104411	BioLegend

Supplemental Table 5. List of ELISA reagents used in the study.

Cytokine/chemokine	Ordering number	Company	Sample dilution
**IL-10**	M1000B<br>Mouse IL-10<br>Quantikine ELISA Kit	BioTechne	1:2<br>(according to manufacturer)
**IL-4**	431107<br>LEGEND MAX Mouse IL-4 Kit	BioLegend	not diluted<br>(1:2 if needed)
**IL-1RA**	MRA00<br>Mouse IL-1RA<br>Quantikine ELISA Kit	BioTechne	not diliuted
**IL-5**	M5000<br>Mouse IL-5<br>Quantikine ELISA Kit	BioTechne	not diluted
**CCL22**	MCC220<br>Mouse CCL22<br>Quantikine ELISA Kit	BioTechne	1:3<br>(according to manufacturer)
**TNFα**	MTA00b<br>Mouse TNFα<br>Quantikine ELISA Kit	BioTechne	not diluted
**IL-6**	M6000B-1<br>Mouse IL-6<br>Quantikine ELISA Kit	BioTechne	not diluted
**CCL5/RANTES**	MMR00<br>Mouse/Rat CCL5<br>Quantikine ELISA Kit	BioTechne	not diluted<br>(1:2 if needed)
**KC1/GROα**	447507<br>Mouse CXCL1<br>LegendMax ELISA Kit	BioLegend	not diluted
