# Supplementary figures and images for: Sphingosine 1-phosphate receptor 1 signaling in macrophages reduces atherosclerosis in LDL receptor–deficient mice
Source: JCI Insight. 2024 Nov 12;9(24):e158127. doi: 10.1172/jci.insight.158127 (PMC11665566; doi:10.1172/jci.insight.158127)

**Main Text – Fig. 6C**

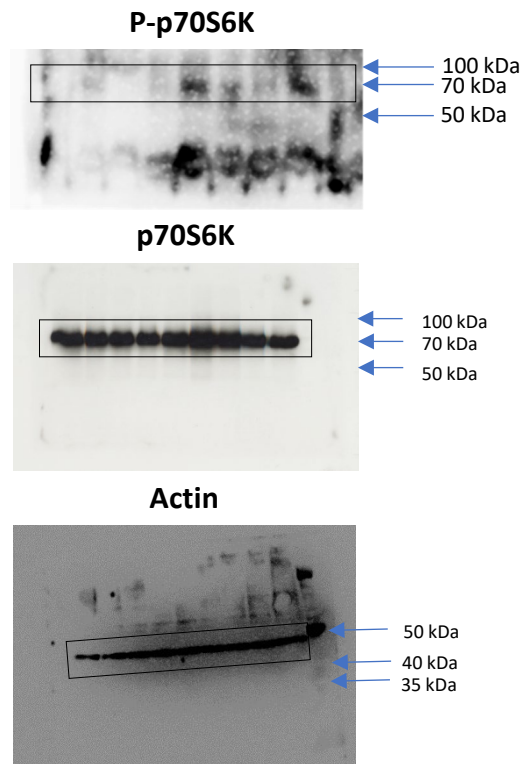

**Supplemental Material – Fig. 1C**

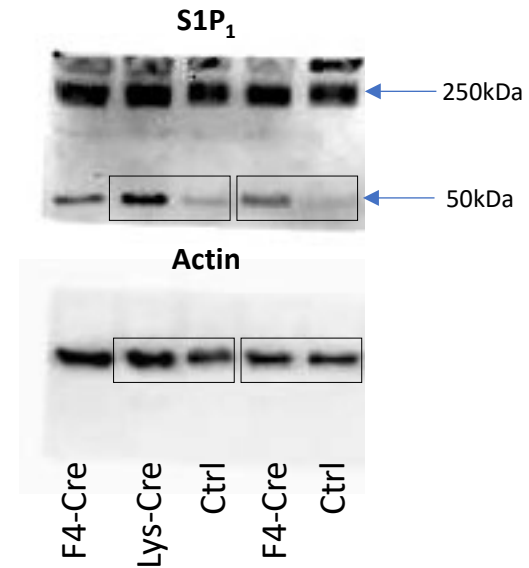

**Supplemental Material – Fig. 9A**

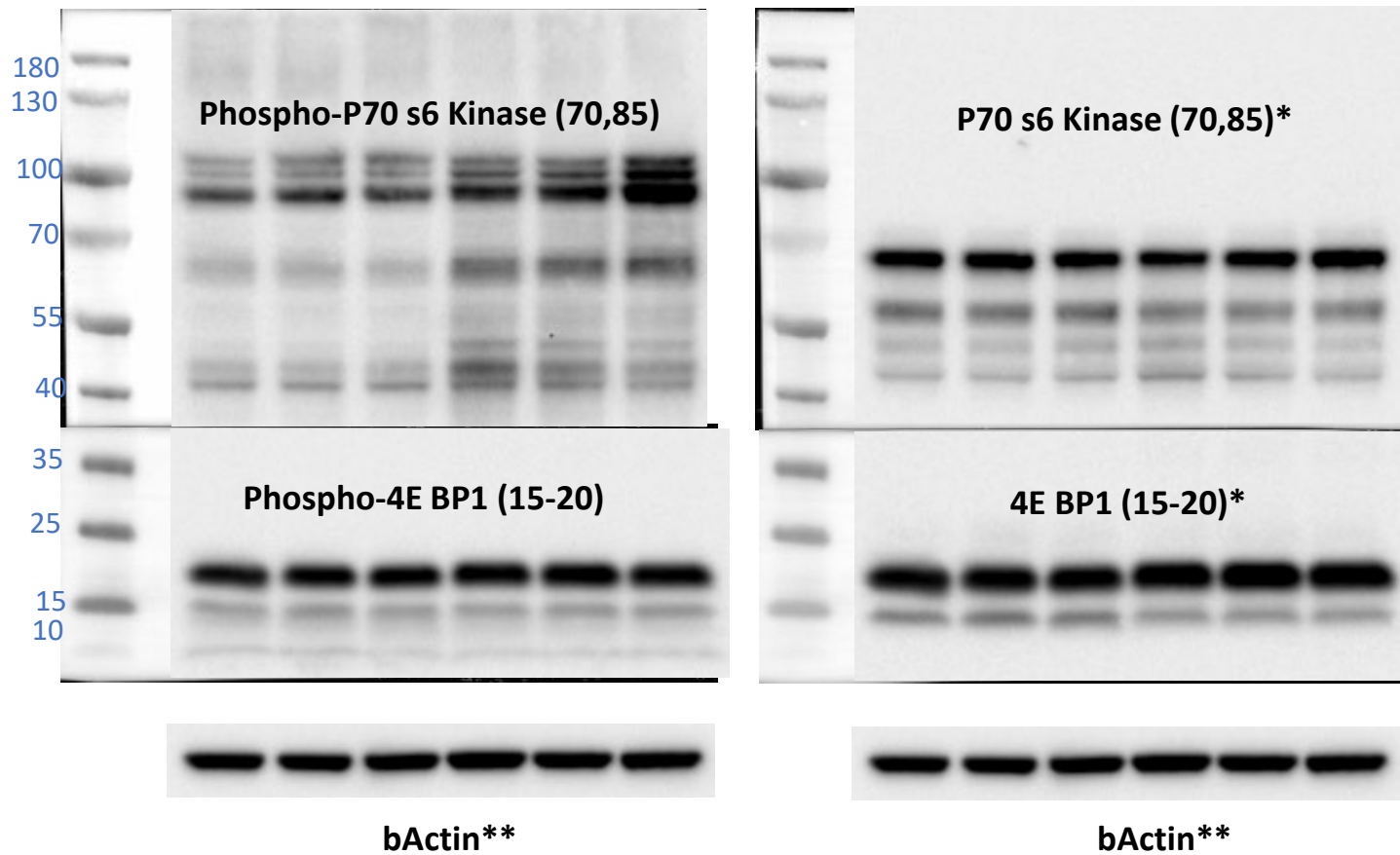

Supplement: Unedited blot and gel images [file jciinsight-9-158127-s009.pdf]
